# Supplementary material for: A knowledge-guided pre-training framework for improving molecular representation learning
Source: Nat Commun. 2023 Nov 21;14:7568. doi: 10.1038/s41467-023-43214-1 (PMC10663446; doi:10.1038/s41467-023-43214-1)
Supplement: Supplementary file 1 — Supplementary Information [file 41467_2023_43214_MOESM1_ESM.pdf]

1 Supplementary notes

1.1 Brief introduction of the downstream molecular property datasets

We employed three benchmarks in our tests to comprehensively evaluate the prediction performance of KPGT.

The first benchmark contains datasets that had been widely used to evaluate the prediction performance of self-supervised learning methods on molecules, including eight classification datasets and three regression datasets. The detailed information for these datasets is listed below:

- BACE is a collection of 1,513 molecules, providing binary labels of molecules which measure whether these molecules can act as the inhibitors of human β-secretase 1 (BACE-1) or not[65].
- BBBP is a binary classification dataset that contains 2,039 molecules, recording whether these molecules can penetrate the blood-brain barrier or not[47].
- SIDER records the adverse drug reactions of 1,427 marked drugs, providing twenty-seven classification tasks[39].
- ClinTox is a collection of 1,478 drugs approved through the US Food and Drug Administration-approved (FDA) and eliminated due to the toxicity issues during clinical trials, providing two binary classification tasks[22].
- ToxCast contains 617 toxicity binary labels for 8,575 molecules obtained by high-throughput screening tests[55].
- Tox21 is a public database containing twelve toxicity binary labels for 7,831 molecules[1].
- Estrogen contains 3,122 molecules with known activities towards the estrogen receptors extracted from the ChEMBL dataset, providing one binary classification task[20].
- MetStab is a dataset measuring the half-life time of 2,267 molecules within an organism, providing one binary classification task[54].
- ESOL is a dataset recording the solubility of 1,128 molecules, providing one regression task[13].
- Lipophilicity is a dataset measuring the molecular membrane permeability and solubility for 4,200 molecules, providing one regression task[20].
- FreeSolv contains the hydration-free energy of 642 molecules in water from both experiments and alchemical-free energy calculation[50].

The second benchmark is the Therapeutics Data Commons (TDC) benchmark, which provides twenty-two datasets measuring the absorption, distribution, metabolism, excretion and toxicity of molecules[30,31]. The detailed information for these datasets from the TDC benchmark is listed below:

- Absorption datasets
  - Caco2 is a collection of 906 molecules with labels measuring the Caco-2 cell effective permeability, providing one regression task[74].
  - HIA is a dataset measuring the activity of human intestinal absorption (HIA) of 578 molecules, providing one binary classification task[26].
  - Pgp contains 1,212 activity labels of P-glycoprotein (Pgp) inhibition, providing one binary classification task[5].
  - Bioav is a collection of 640 molecules with labels measuring the activity of bioavailability, providing one binary classification task[45].
  - Lipo is a dataset measuring the molecular membrane permeability and solubility for 4,200 molecules, providing one regression task[20].

  - AqSol contains 9,982 labels measuring the solubility of molecules, providing one regression task[63].
- Distribution datasets
  - BBB is a dataset measuring the activity of the blood-brain barrier (BBB) of 1,975 molecules, providing one binary classification dataset[47].
  - PPBR is a dataset measuring the human plasma protein binding rates (PPBRs) of 1,614 molecules, providing one regression dataset[77].
  - VDss is a collection of 1,130 with labels measuring the volume of distribution at steady state (VDss)[44].
- Metabolism datasets
  - CYP2C9$_{inh}$ is a dataset measuring the CYP2C9 inhibition of 12,092 molecules, providing one binary classification task[70].
  - CYP2D6$_{inh}$ is a dataset measuring the CYP2D6 inhibition of 13,130 molecules, providing one binary classification task[70].
  - CYP3A4$_{inh}$ is a dataset measuring the CYP3A4 inhibition of 12,328 molecules, providing one binary classification task[70].
  - CYP2C9$_{sub}$ contains 666 molecules with labels measuring whether each molecule is a substrate of the CYP2C9 or not, providing one binary classification task[9].
  - CYP2D6$_{sub}$ contains 664 molecules with labels measuring whether each molecule is a substrate of the CYP2D6 or not, providing one binary classification task[9].
  - CYP3A4$_{sub}$ contains 667 molecules with labels measuring whether each molecule is a substrate of the CYP3A4 or not, providing one binary classification task[9].
- Excretion datasets
  - HalfLife measures the half-life duration of 667 molecules, providing one regression task[52].
  - CL-Hepa measures the activity of hepatocyte clearance of 1,020 molecules, providing one regression task[14].
  - CL-Micro measures the activity of microsome clearance of 1,102 molecules, providing one regression task[14].
- Toxicity datasets
  - LD50 is a collection of 7,385 molecules with labels measuring their acute toxicity, providing one regression dataset[91].
  - hERG is a dataset containing 648 molecules measuring whether each molecule is a human ether-a-go-go related gene (hERG) blocker or not, providing one classification dataset[75].
  - Ames is a dataset containing 7,255 molecules measuring whether each molecule is mutagenic or not, providing one binary classification dataset[81].
  - DILI is a dataset containing 475 molecules measuring whether each molecule can cause liver injury or not, providing one binary classification dataset[84].

The third benchmark is the MoleculeACE benchmark[67], which contains thirty datasets of molecules with activity cliffs and measures bio-activity of molecules against thirty targets (e.g., androgen receptor, canabinoid receptor 1 and coagulation factor X) collected from the ChEMBL29 dataset[21]. Activity cliffs are pairs of molecules that are highly similar in their structures but exhibit large differences in potency, thus posing a great challenge for the prediction. Each dataset in the benchmark was split into training and test sets with a ratio of 8:2. Please refer to[67] for more detailed information about these datasets.

1.2 Brief introduction of the baseline methods

In the first benchmark tests, we employed fourteen self-supervised learning baseline methods. The detailed information for these methods is listed below:

- Infomax pre-trains a graph isomorphism network (GIN) through maximizing mutual information between node representations and the corresponding graph representations[72].
- Edgepred pre-trains a GIN through learning to predict the connectivity of node pairs[24].
- Attribute Masking (Masking) pre-trains a GIN through learning to predict the masked features of nodes[28].
- Context Prediction (Contextpred) pre-trains a GIN through learning to predict the surrounding graph structures of subgraphs[28].
- Supervised+Attribute Masking (Masking$_{sup}$) pre-trains a GIN through the Masking strategy following a supervised graph-level bio-activities prediction task[28].
- Supervised+Context Prediction (Contextpred$_{sup}$) pre-trains a GIN using the Contextpred strategy and subsequently pre-trains with a supervised graph-level bio-activities prediction task[28].
- Supervised+Infomax (Infomax$_{sup}$) pre-trains a GIN using the Contextpred strategy and subsequently pre-trains with a supervised graph-level bio-activities prediction task[28].
- Supervised+Edgepred (Edgepred$_{sup}$) pre-trains a GIN using the Contextpred strategy and subsequently pre-trains with a supervised graph-level bio-activities prediction task[28].
- GraphLoG pre-trains a GIN through learning the hierarchical prototypes upon graph embeddings[83].
- GraphCL pre-trains a GIN through aligning the original graphs and the corresponding augmented ones generated by strategies including node dropping, edge perturbation, attribute masking and subgraph derivation in latent space via contrastive learning[87].
- JOAO is an improved version of GraphCL, which adaptively and dynamically selects data augmentations when performing contrastive learning[86].
- GROVER conducts pre-training of a message-passing transformer by incorporating both node-level and graph-level tasks. The node-level task involves predicting masked subgraphs, while the graph-level task focuses on predicting molecular motifs.[58]. Note that 200 molecular descriptors are concatenated with molecular representations produced by GROVER for deriving final prediction.
- 3DInfomax pre-trains a principle neighbor aggregation network (PNA) via maximizing the mutual information between the 3D graph representations and the corresponding 2D graph representations[64].
- GraphMVP pre-trains a GIN via leveraging the correspondence and consistency between 2D molecular structures and 3D geometric views[42].
- ImageMol pre-trains a ResNet-18 model by leveraging the local and global structural characteristics of molecules from pixels of molecular images[89].
- MolFormer pre-trains a linear attention transformer via a masking language model based approach based on the SMILES representations of molecules[59].
- GEM introduces a geometry-based graph neural network architecture as well as several dedicated geometry-level self-supervised learning strategies to learn the molecular geometry knowledge[16].
- GraphMAE introduces a masked graph autoencoder that conducts pre-training with a feature reconstruction strategy[27].
- MoleBERT integrates a node-level pre-training strategy named masked atoms modeling and a triplet masked contrastive learning strategy for graph-level pre-training[79].

The TDC benchmarking platform provides twenty-eight methods, including six graph neural network-based methods (i.e., AttentiveFP[80], GCN[38], SimGCN, QuGIN[82]), Chemprop[85] and Chemprop-RDKit[85],

two convolutional neural networks, including MolMapNet-D[62] and CNN[32], two self-supervised learning based methods (i.e., ContextPred[28] and AttrMasking[28]), five deep neural network based methods, including RDKit2D + MLP[32], Morgan + MLP[32], NeuralFP[40], Lantern RADR Deep Neural Network (LRDNN)[8] and Lantern RADR Ensemble (LRE)[8], one automated machine learning method MACCS keys + autoML[60], twelve methods based on molecular descriptors and fingerprints (i.e., XGBoost_Li[29], Euclia ML model[8], ZairaChem[69], Basic ML[3], BaseBoosting[29], BaseBoosting KyQVZ6b2 (BaseBoosting K)[29], Lantern RADR Logistic Regression (LRLR)[8], Lantern RADR Random Forest (LRRF)[8], RFStacker[29], ColorRefinement + Weighted Ensemble LGBM (CR+WE LGBM)[8], Voting Regressor[8] and Random Forest[29]).

The MoleculeACE benchmarking platform provides twenty-four baseline methods, including sixteen machine learning methods, i.e., combinations of four mahcine learning models (including support vector machine (SVM)[11], random forest (RF)[4], gradient boosting machine (GBM)[18]) and k-nearest neighbor (KNN)[17]) and four molecular fingerprint or descriptor based methods (including ECFP[56], MACCS[15], PHYSCHEM[73] and WHIM[68]), four graph neural network based methods, i.e., message passing neural network (MPNN)[23], graph attention network (GAT)[71], graph convolutional network (GCN)[38] and attentive fingerprint (AFP)[80], one CNN[37] model based on SMILES, and two self-supervised learning methods based on SMILES, i.e., the long short-term memory (LSTM)[51,61] and the transformer model[10].

2 Supplementary Figures

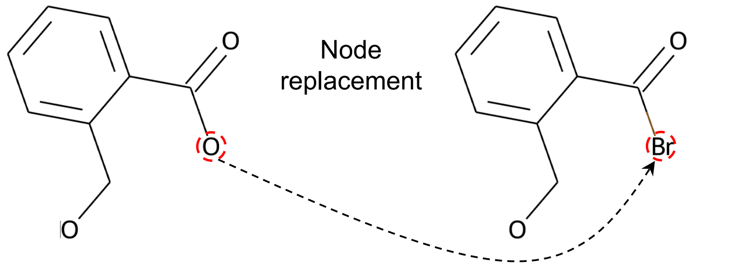

Property	Original molecule	Modified molecule
LogP	0.888	1.714
SA	57.5	37.3
MolWt	152	215

Supplementary Fig. 1: A small modification of a molecule can lead to great changes in its properties.

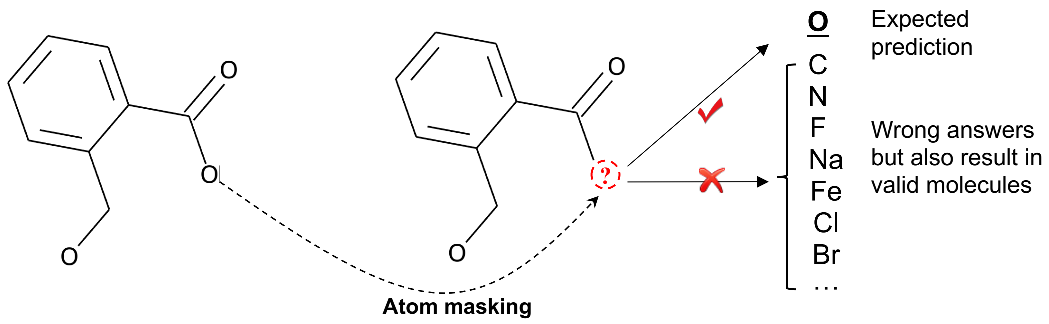

Supplementary Fig. 2: The dependence between the masked node and its neighboring node is weak.

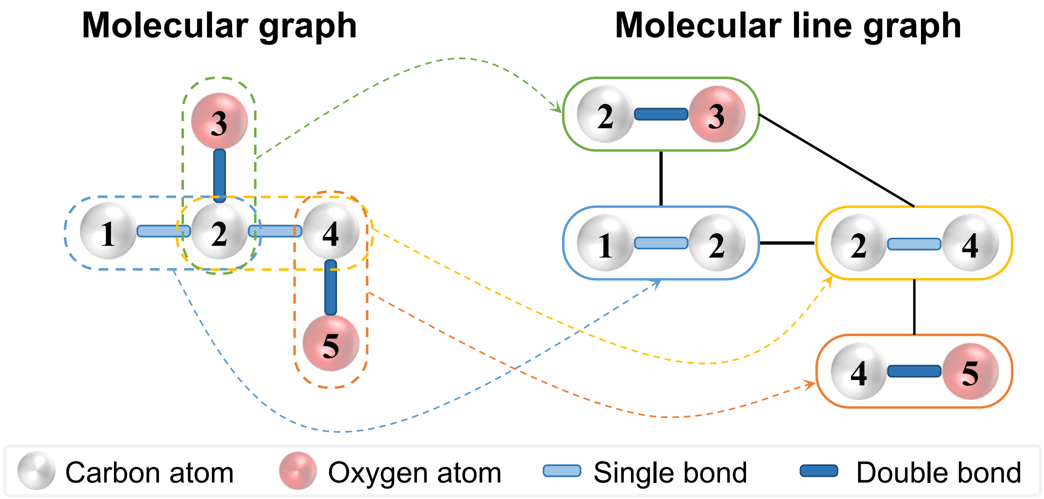

Supplementary Fig. 3: An illustrative example of the transformation of a molecular graph to a molecular line graph.

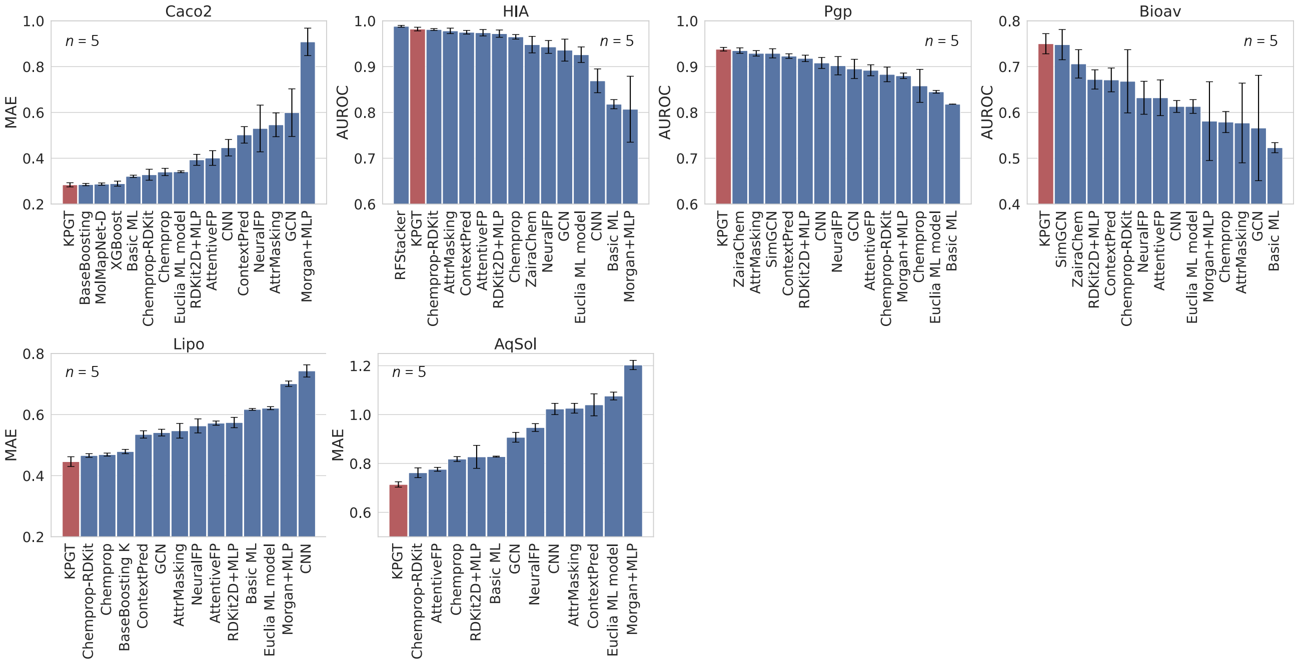

Supplementary Fig. 4: Prediction performance of KPGT and baseline methods on the datasets measuring molecular absorption from TDC. The results were reported based on five independent runs. Source data are provided as a Source Data file.

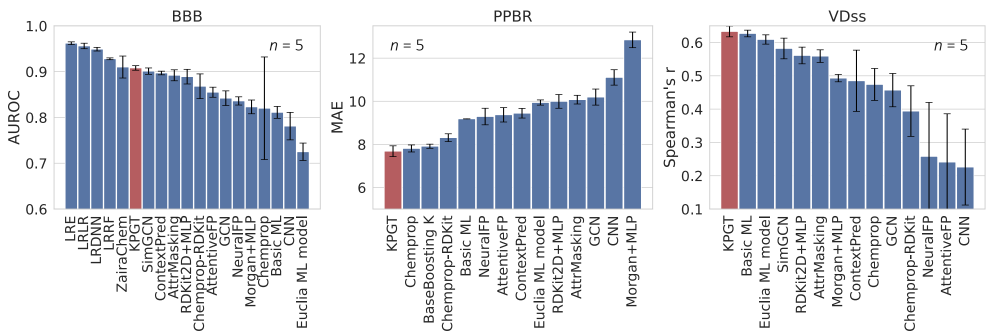

Supplementary Fig. 5: Prediction performance of KPGT and baseline methods on the datasets measuring molecular distribution from TDC. The results were reported based on five independent runs. Source data are provided as a Source Data file.

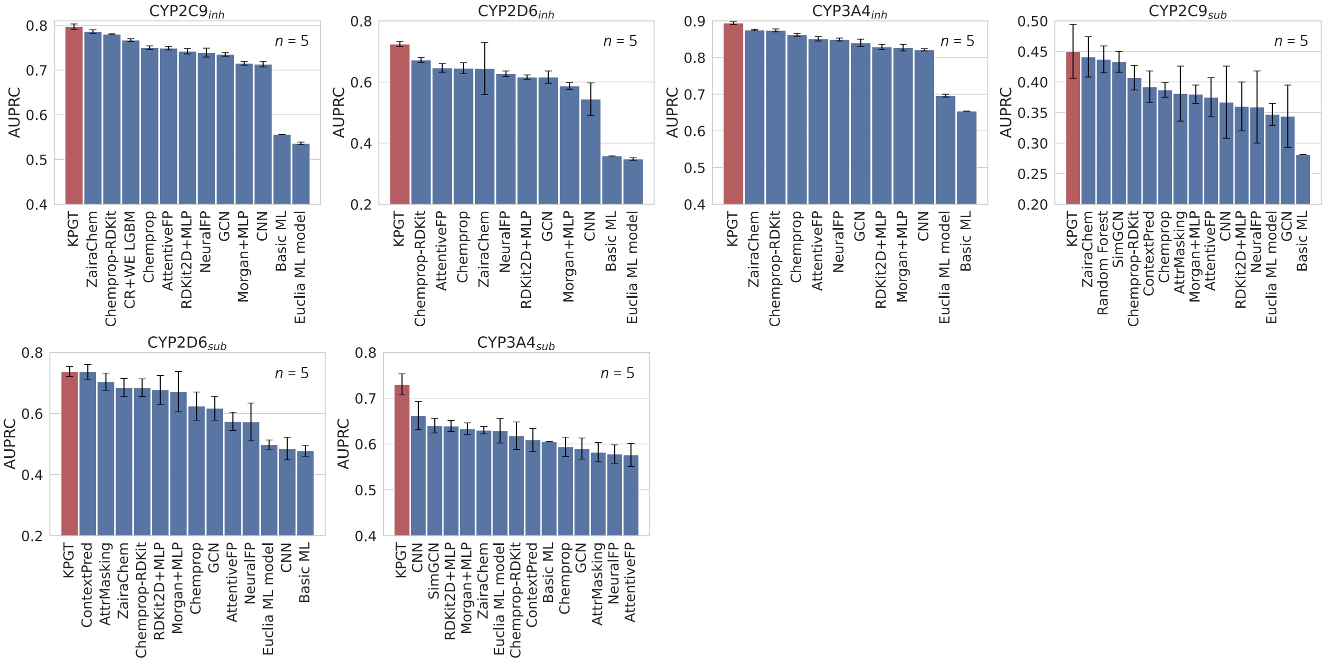

Supplementary Fig. 6: Prediction performance of KPGT and baseline methods on the datasets measuring molecular metabolism from TDC. The results were reported based on five independent runs. Source data are provided as a Source Data file.

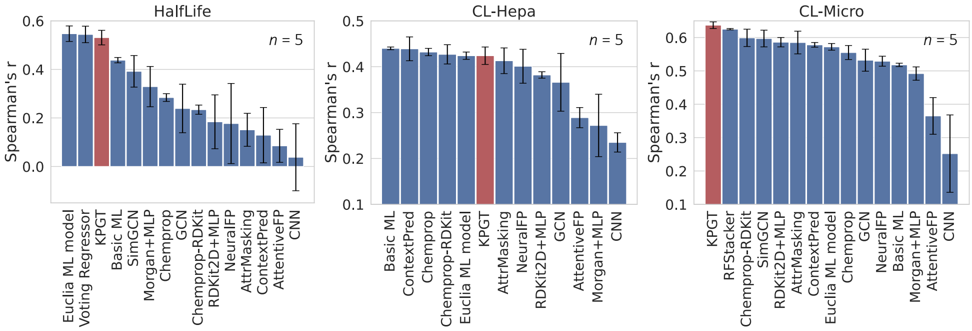

Supplementary Fig. 7: Prediction performance of KPGT and baseline methods on the datasets measuring molecular excretion from TDC. The results were reported based on five independent runs. Source data are provided as a Source Data file.

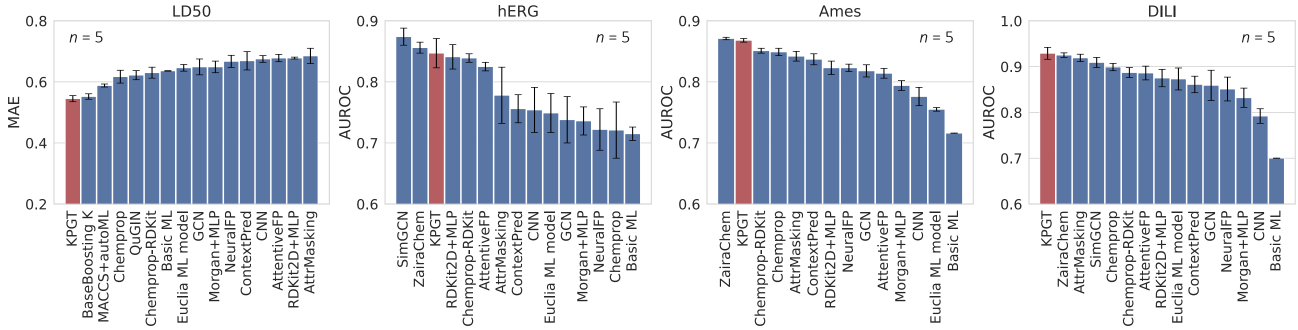

Supplementary Fig. 8: Prediction performance of KPGT and baseline methods on the datasets measuring molecular toxicity from TDC. The results were reported based on five independent runs. Source data are provided as a Source Data file.

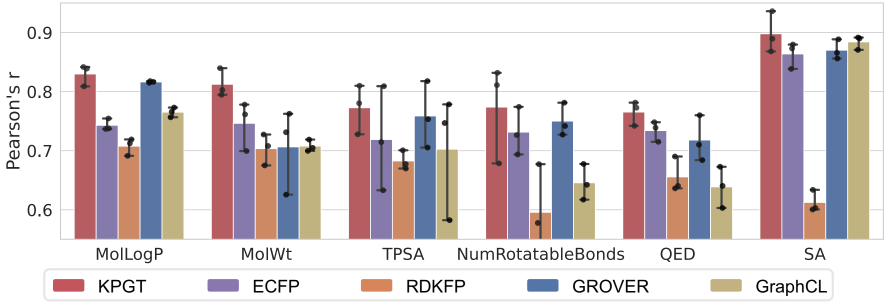

Supplementary Fig. 9: The Spearman's correlation coefficient (Spearman's r) between five descriptors (i.e., MolLogP, MolWt, TPSA, NumRotatableBonds, QED, and SA) of 200 target molecules and those of their closest molecules derived based on the fingerprints provided by KPGT and baseline methods, respectively. The results were based on three independent runs. Source data are provided as a Source Data file.

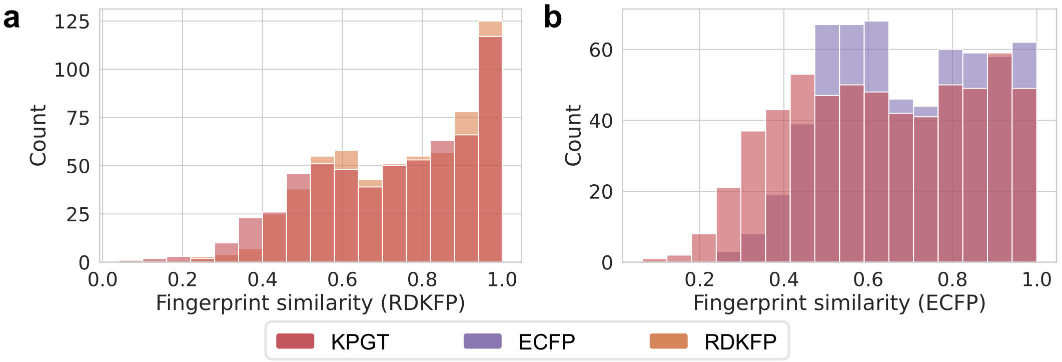

Supplementary Fig. 10: **a** The distributions of the structural similarity (measured by RDKFP) between target molecules and the corresponding molecules queried by KPGT and RDKFP. **b** The distributions of the structural similarity (measured by ECFP) between target molecules and the corresponding molecules queried by KPGT and ECFP. As we used RDKFP/ECFP for both querying molecules and measuring structural similarity, these results provided upper bounds for this test. Source data are provided as a Source Data file.

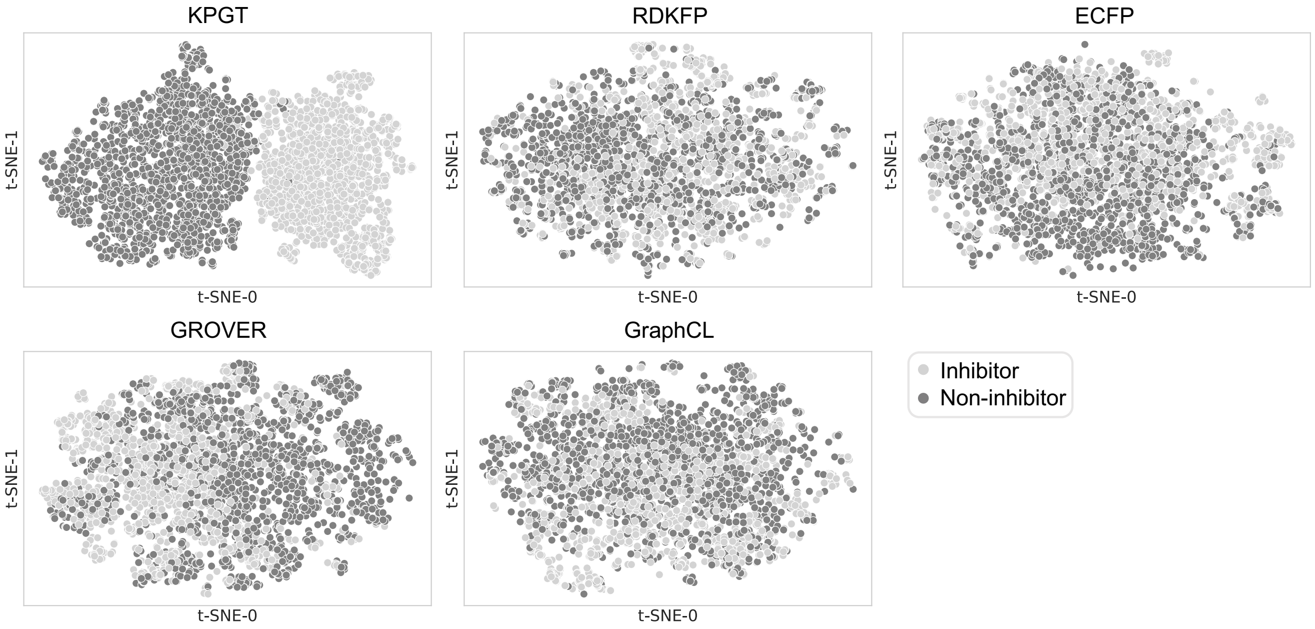

Supplementary Fig. 11: Visualization of the distributions of inhibitor and non-inhibitor molecules from the test set of the CYP3A4 dataset for KPGT, ECFP, RDKFP, GROVER, and GraphCL, using t-distributed stochastic neighbor embedding (t-SNE). Source data are provided as a Source Data file.

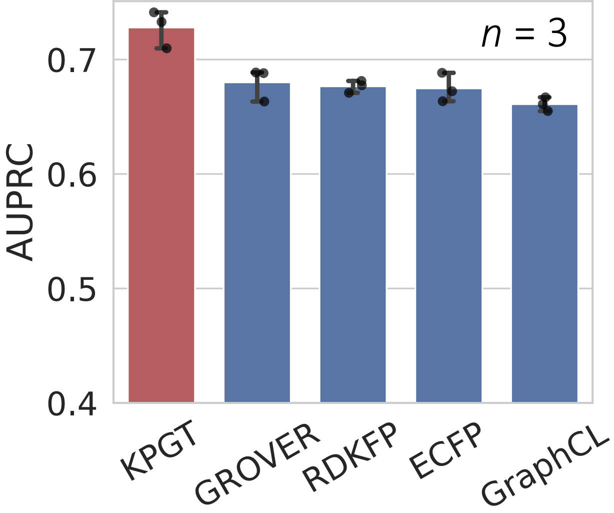

Supplementary Fig. 12: Prediction performance of KPGT and baseline methods on the test set with only activity cliff molecules of the CYP3A4 dataset, measured in terms of AUPRC. The results were based on three independent runs. Source data are provided as a Source Data file.

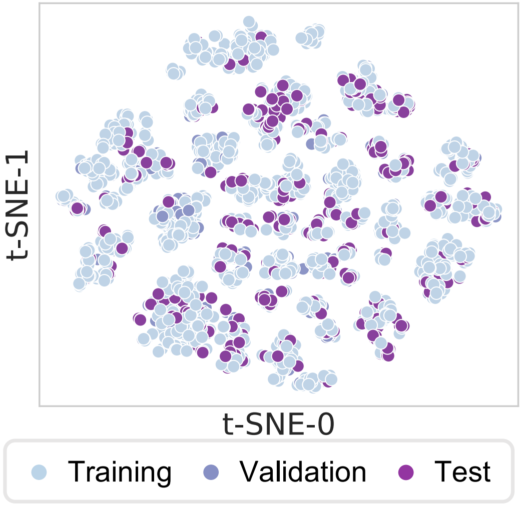

Supplementary Fig. 13: Visualization of the molecular ECFPs via t-SNE under the scaffold splitting scenario. Source data are provided as a Source Data file. Source data are provided as a Source Data file.

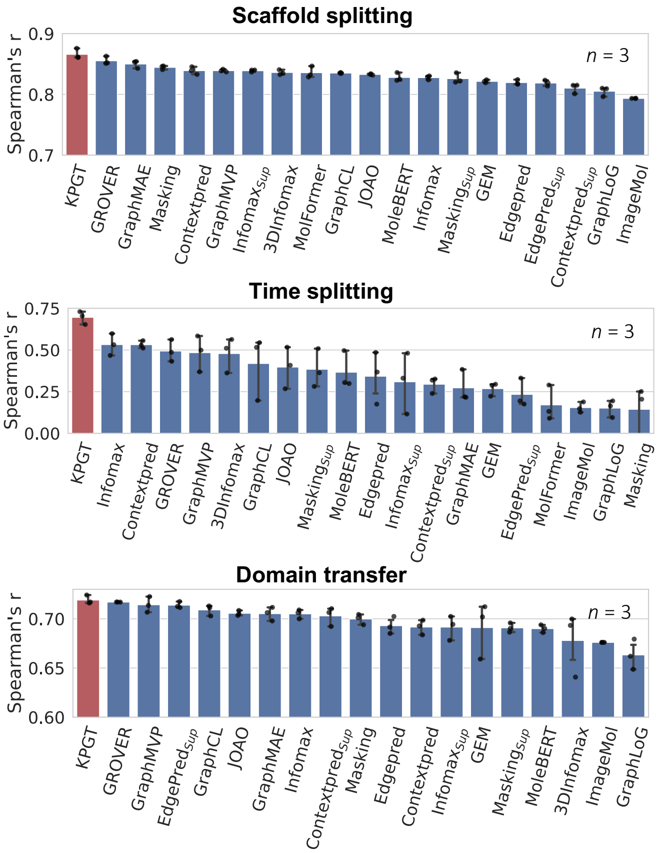

Supplementary Fig. 14: Prediction performance of KPGT and baseline methods on the HPK1 dataset under the scaffold splitting, time splitting, and domain transfer scenarios, measured in terms of Spearman's r. Source data are provided as a Source Data file.

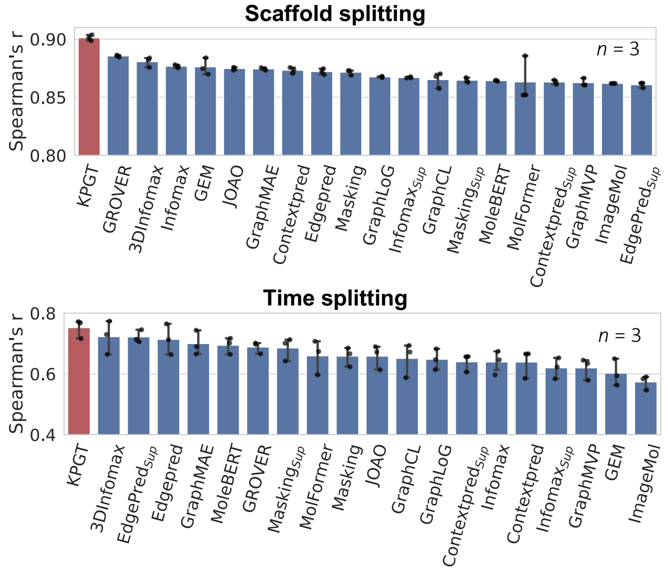

Supplementary Fig. 15: Prediction performance of KPGT and baseline methods on the FGFR1 dataset under the scaffold splitting, time splitting, and domain transfer scenarios, measured in terms of Spearman's r. Source data are provided as a Source Data file.

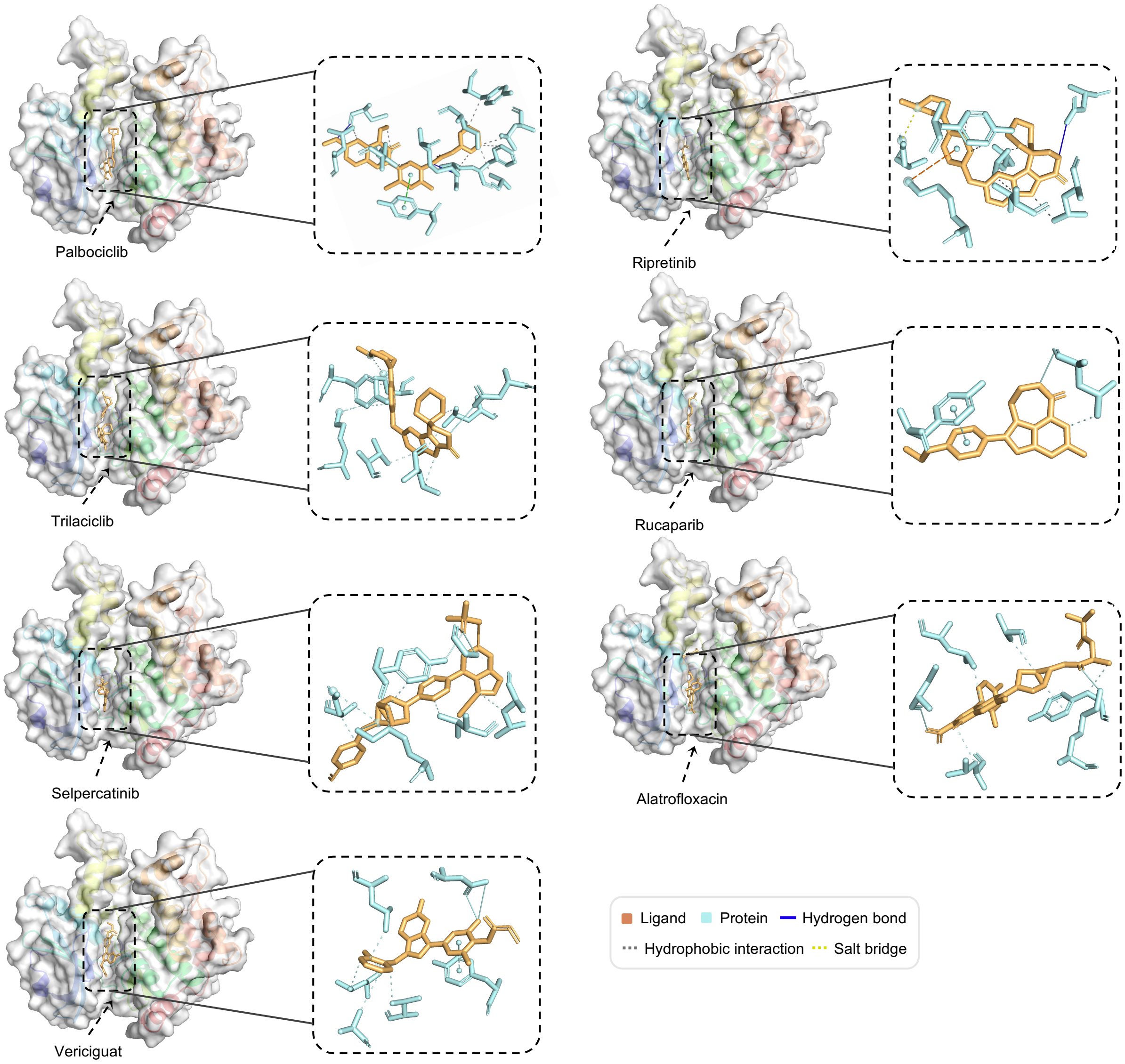

Supplementary Fig. 16: Docking results of previously unreported potential HPK1 inhibitory molecules identified by KPGT. The interactions between molecules and HPK1 were profiled by PLIP[2]. The protein-ligand structure (PDB ID: 7SIU[46]) was utilized as a reference for the identification of the binding pocket.

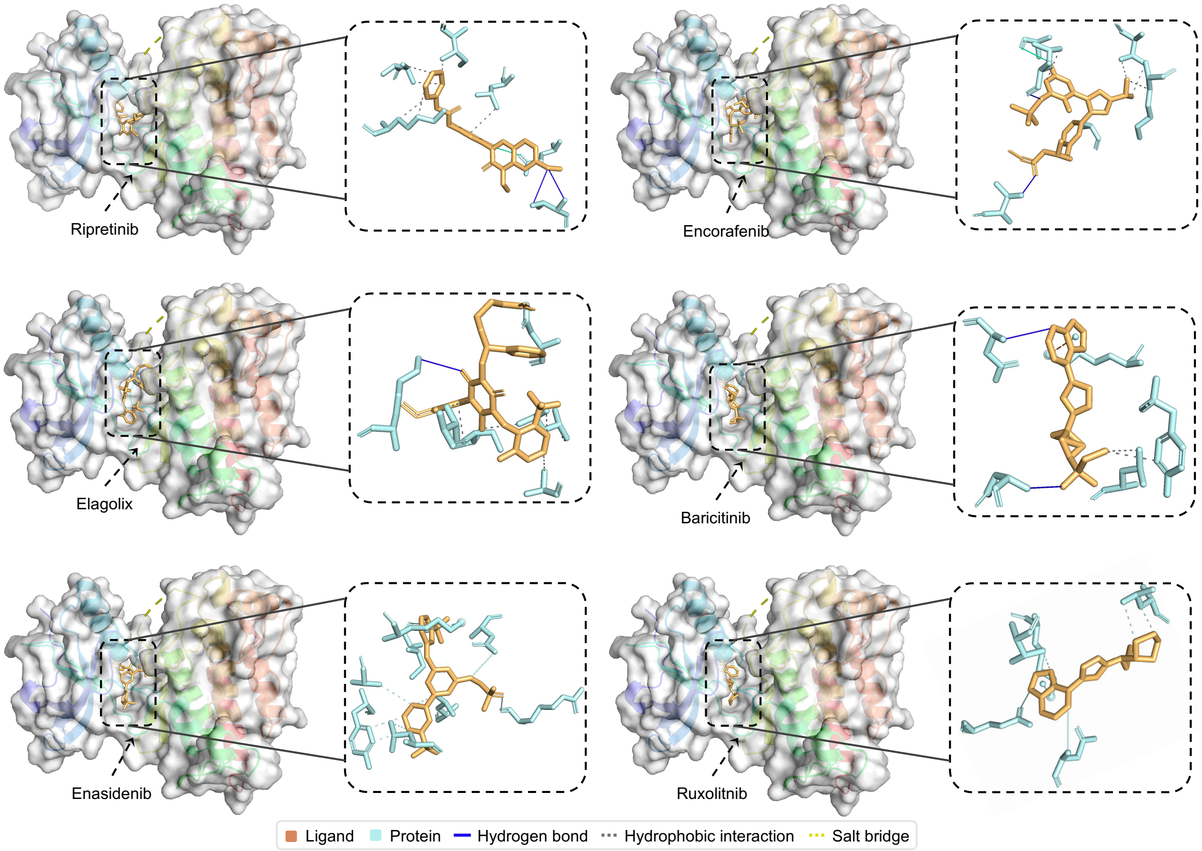

Supplementary Fig. 17: Docking results of previously unreported potential FGFR1 inhibitory molecules identified by KPGT. The interactions between molecules and FGFR1 were profiled by PLIP[2]. The protein-ligand structure (PDB ID: 5A4C[46]) was utilized as a reference for the identification of the binding pocket.

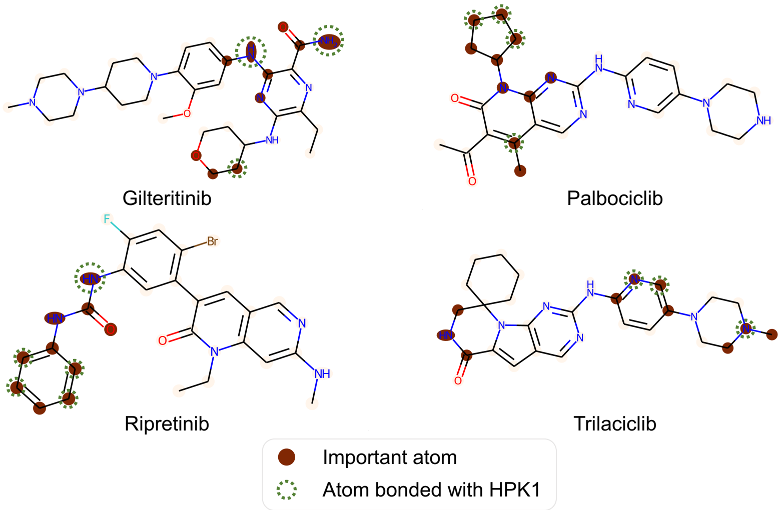

Supplementary Fig. 18: SubgraphX[88] identifies atoms that form interactions with HPK1 profiles by PLIP[2].

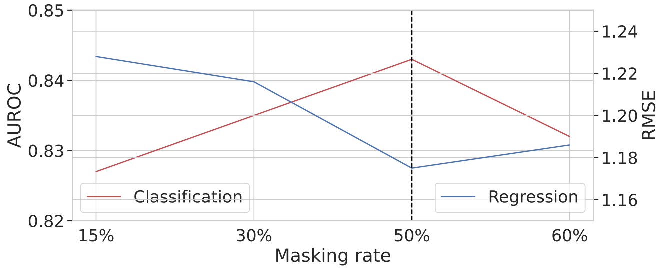

Supplementary Fig. 19: Prediction performance of KPGT given different masking rates on the classification and regression datasets from the first benchmarking test in the Results section, measured in terms of AUROC and RMSE, respectively. Source data are provided as a Source Data file.

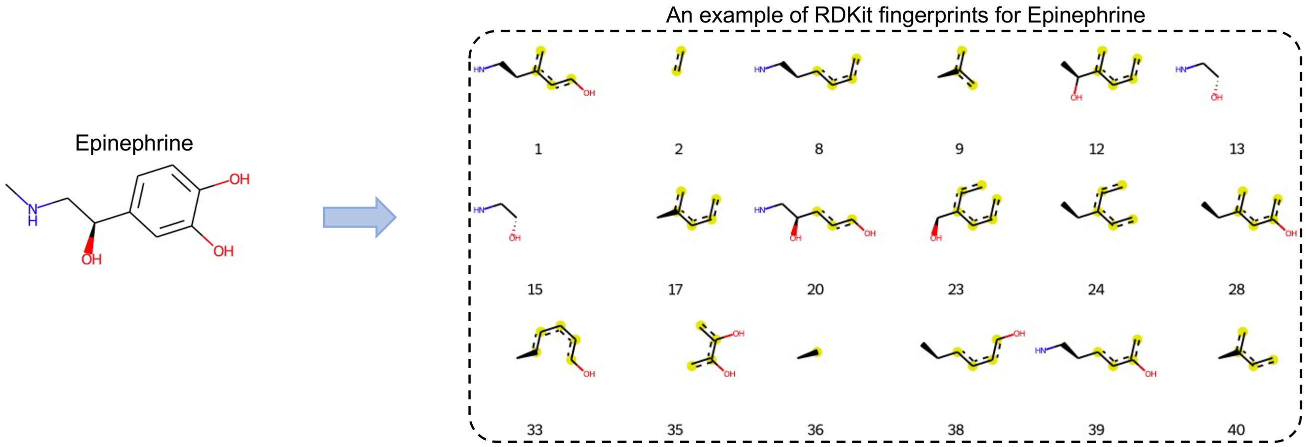

Supplementary Fig. 20: An example of RDKit fingerprints of epinephrine. The numbers below the substructures indicate the positions of the corresponding fingerprints in the bit string.

3 Supplementary Tables

Supplementary Table 1: Atom features used in the molecular line graph.

Feature	Size	Description
Atom type	101	type of atoms (e.g., C, N, O), by atomic number (one-hot)
Degree	12	number of bonds the atom is involved in (one-hot)
Formal charge	1	electronic charge assigned to the atom (integer)
Chiral center	1	whether the atom is charity center (integer)
Chirality type	2	R or S (one-hot)
Number of H	6	number of bonded hydrogen atoms (one-hot)
Atomic mass	1	mass of the atom, divided by 100 (float)
Aromaticity	1	whether this atom is part of an aromatic system (bionary)
Radical electrons	6	number of radical electrons (one-hot)
Hybridization	6	sp, sp^2, sp^3, sp^3d, sp^3d^2 or unknown (one-hot)

Supplementary Table 2: Bond features used in the molecular line graph.

Feature	Size	Description
Bond type	5	type of bonds (i.e., single, double, triple, aromatic or unknown) (one-hot)
Stereo	7	StereoNone, StereoAny, StereoZ, StereoE, StereoCis, StereoTrans or unknown(one-hot)
In ring	1	whether the bond is part of a ring (binary)
Conjugated	1	whether the bond is conjugated (binary)

Supplementary Table 3: The hyper-parameter settings of KPGT in the pre-training and finetuning process.

Hyper-parameter	Pre-training	Finetuning
Hidden size	768	768
Number of layers	12	12
Number of attention heads	12	12
Dropout rate	0.1	[0, 0.05, 0.1, 0.2]
Batch size	1024	32
Learning rate	$2e^{-4}$	[$1e^{-6}$,$3e^{-6}$,$1e^{-5}$,$3e^{-5}$]
Learning rate decay	polynomial	polynomial
Weight decay	$1e^{-6}$	[0,$1e^{-6}$,$1e^{-4}$]
Gradient clipping	5.0	5.0
Adam β_1	0.9	0.9
Adam β_2	0.999	0.999
Predictor hidden size	NA	256
Predictor layer	NA	2
Masking rate	0.5	NA
LLRD decay rate	NA	[0.95, 0.9, 0.85, 0.8]
ReInit top-n layer	NA	[1,2,3,4]
FLAG step size	NA	[$5e^{-3}$, $3e^{-3}$, $1e^{-2}$, $1e^{-3}$]
L^2-SP weight	NA	[$1e^{-1}$,$1e^{-2}$,$1e^{-3}$,$1e^{-4}$]

Supplementary Table 4: The AUROC performance of different methods on the classification datasets under the feature extraction setting. AVG represents the averaging results over all the datasets. The numbers in brackets are the standard deviations over three independent runs. The best result for each dataset is marked in bold and the second-best result is underlined.

Method	Classification dataset								
	BACE	BBBP	ClinTox	SIDER	Estrogen	MetStab	Tox21	ToxCast	**AVG**
Infomax[72]	0.825 (0.033)	0.833 (0.017)	0.637 (0.027)	0.573 (0.016)	0.878 (0.061)	0.763 (0.044)	0.764 (0.038)	0.662 (0.010)	0.742
Edgepred[24]	0.807 (0.014)	0.823 (0.011)	0.637 (0.059)	0.545 (0.019)	0.851 (0.070)	0.792 (0.064)	0.702 (0.040)	0.620 (0.004)	0.722
Masking[28]	0.739 (0.059)	0.823 (0.031)	0.564 (0.089)	0.555 (0.032)	0.805 (0.075)	0.588 (0.102)	0.722 (0.017)	0.591 (0.023)	0.673
Contextpred[28]	0.822 (0.053)	0.875 (0.009)	0.607 (0.062)	0.585 (0.025)	0.872 (0.059)	0.791 (0.078)	0.757 (0.028)	0.671 (0.009)	0.748
Infomax$_{sup}$[28]	0.790 (0.041)	0.854 (0.022)	0.739 (0.045)	0.611 (0.018)	0.855 (0.066)	0.772 (0.091)	0.816 (0.012)	0.709 (0.013)	0.768
Edgepred$_{sup}$[28]	0.815 (0.040)	0.874 (0.032)	0.709 (0.068)	0.619 (0.005)	0.862 (0.044)	0.755 (0.087)	0.812 (0.010)	0.707 (0.011)	0.769
Masking$_{sup}$[28]	0.818 (0.039)	0.849 (0.024)	0.792 (0.043)	0.611 (0.003)	0.888 (0.038)	0.819 (0.054)	0.828 (0.013)	0.685 (0.013)	0.786
Contextpred$_{sup}$[28]	0.831 (0.018)	0.852 (0.039)	0.765 (0.061)	0.612 (0.026)	0.871 (0.051)	0.807 (0.039)	0.829 (0.026)	0.687 (0.008)	0.782
GraphLoG[83]	0.766 (0.040)	0.799 (0.025)	0.526 (0.101)	0.583 (0.016)	0.867 (0.071)	0.747 (0.063)	0.691 (0.041)	0.608 (0.011)	0.698
GraphCL[87]	0.802 (0.045)	0.836 (0.021)	0.663 (0.018)	0.576 (0.011)	0.864 (0.050)	0.779 (0.062)	0.758 (0.036)	0.639 (0.012)	0.740
JOAO[86]	0.834 (0.033)	0.854 (0.023)	0.747 (0.039)	0.612 (0.007)	0.854 (0.061)	0.800 (0.062)	0.801 (0.023)	0.653 (0.014)	0.769
GROVER[58]	0.809 (0.046)	0.885 (0.009)	0.809 (0.048)	0.591 (0.030)	0.870 (0.048)	0.818 (0.056)	0.827 (0.019)	0.708 (0.015)	0.790
3DInfomax[64]	0.810 (0.044)	0.806 (0.037)	0.743 (0.014)	0.585 (0.021)	0.852 (0.054)	0.784 (0.072)	0.738 (0.021)	0.609 (0.021)	0.741
GraphMVP[42]	0.741 (0.012)	0.836 (0.028)	0.696 (0.051)	0.563 (0.023)	0.833 (0.071)	0.669 (0.057)	0.778 (0.022)	0.645 (0.006)	0.720
MolFormer[59]	0.792 (0.013)	0.866 (0.024)	0.802 (0.053)	0.578 (0.012)	0.805 (0.081)	0.654 (0.061)	0.764 (0.022)	0.686 (0.006)	0.743
ImageMol[89]	0.770 (0.053)	0.833 (0.008)	0.793 (0.025)	0.608 (0.007)	0.862 (0.056)	0.794 (0.043)	0.801 (0.021)	0.678 (0.003)	0.767
GEM[16]	0.827 (0.029)	0.882 (0.035)	**0.869** (0.028)	0.617 (0.013)	0.876 (0.046)	0.828 (0.056)	0.811 (0.018)	0.723 (0.014)	0.804
GraphMAE[27]	0.725 (0.009)	0.832 (0.023)	0.569 (0.068)	0.545 (0.005)	0.815 (0.102)	0.735 (0.022)	0.712 (0.020)	0.637 (0.007)	0.696
MoleBERT[79]	0.827 (0.021)	0.856 (0.022)	0.758 (0.055)	0.623 (0.006)	0.868 (0.039)	0.767 (0.037)	0.825 (0.012)	0.704 (0.009)	0.779
KPGT	**0.868** (0.011)	**0.896** (0.010)	0.856 (0.028)	**0.644** (0.018)	**0.890** (0.048)	**0.885** (0.047)	**0.838** (0.020)	**0.727** (0.016)	**0.825**

Supplementary Table 5: The RMSE performance of different methods on the regression datasets under the feature extraction setting. AVG represents the averaging results over all the datasets. The numbers in brackets are the standard deviations over three independent runs. The best result for each dataset is marked in bold and the second-best result is underlined.

	FreeSolv	ESOL	Lipo	**AVG**
Infomax[72]	$4.119_{(0.974)}$	$1.462_{(0.076)}$	$0.978_{(0.076)}$	2.186
Edgepred[24]	$3.849_{(0.950)}$	$2.272_{(0.213)}$	$1.030_{(0.024)}$	2.384
Masking[28]	$3.646_{(0.947)}$	$2.100_{(0.040)}$	$1.063_{(0.028)}$	2.270
Contextpred[28]	$3.141_{(0.905)}$	$1.349_{(0.069)}$	$0.969_{(0.076)}$	1.820
Infomax$_{sup}$[28]	$3.315_{(1.127)}$	$1.333_{(0.045)}$	$0.905_{(0.036)}$	1.851
Edgepred$_{sup}$[28]	$3.537_{(1.000)}$	$1.259_{(0.120)}$	$0.902_{(0.008)}$	1.899
Masking$_{sup}$[28]	$3.210_{(0.876)}$	$1.387_{(0.007)}$	$\underline{0.725}_{(0.033)}$	1.774
Contextpred$_{sup}$[28]	$3.105_{(0.701)}$	$1.477_{(0.038)}$	$0.754_{(0.032)}$	1.779
GraphLoG[83]	$4.174_{(1.077)}$	$2.335_{(0.073)}$	$1.104_{(0.024)}$	2.537
GraphCL[87]	$4.014_{(1.361)}$	$1.835_{(0.111)}$	$0.945_{(0.024)}$	2.264
JOAO[86]	$3.466_{(1.114)}$	$1.771_{(0.053)}$	$0.933_{(0.027)}$	2.056
GROVER[58]	$2.991_{(1.052)}$	$0.928_{(0.027)}$	$0.752_{(0.010)}$	1.557
3DInfomax[64]	$2.919_{(0.243)}$	$1.906_{(0.246)}$	$1.045_{(0.040)}$	1.957
GraphMVP[42]	$2.532_{(0.247)}$	$1.937_{(0.147)}$	$0.990_{(0.024)}$	1.819
MolFormer[59]	$\mathbf{2.246}_{(0.483)}$	$0.930_{(0.072)}$	$0.763_{(0.024)}$	$\underline{1.313}$
ImageMol[89]	$3.051_{(0.618)}$	$2.194_{(0.963)}$	$0.890_{(0.026)}$	2.082
GEM[16]	$2.339_{(0.639)}$	$\underline{0.886}_{(0.029)}$	$0.796_{(0.035)}$	1.340
GraphMAE[27]	$3.782_{(0.691)}$	$2.233_{(0.049)}$	$1.070_{(0.010)}$	2.362
MoleBERT[79]	$3.189_{(0.766)}$	$1.568_{(0.084)}$	$0.829_{(0.025)}$	1.862
KPGT	$\underline{2.314}_{(0.841)}$	$\mathbf{0.848}_{(0.103)}$	$\mathbf{0.656}_{(0.023)}$	**1.273**

Supplementary Table 6: The AUROC performance of different methods on the classification datasets under the transfer learning setting. AVG represents the averaging results over all the datasets. The numbers in brackets are the standard deviations over three independent runs. The best result for each dataset is marked in bold and the second-best result is underlined.

Method	Classification dataset								
	BACE	BBBP	ClinTox	SIDER	Estrogen	MetStab	Tox21	ToxCast	**AVG**
Infomax[72]	$0.839_{(0.008)}$	$0.840_{(0.026)}$	$0.661_{(0.026)}$	$0.616_{(0.024)}$	$0.888_{(0.056)}$	$0.837_{(0.066)}$	$0.816_{(0.021)}$	$0.690_{(0.012)}$	0.773
Edgepred[24]	$0.817_{(0.034)}$	$0.873_{(0.016)}$	$0.730_{(0.017)}$	$0.603_{(0.025)}$	$0.881_{(0.061)}$	$0.844_{(0.054)}$	$0.818_{(0.025)}$	$0.712_{(0.011)}$	0.785
Masking[28]	$0.823_{(0.004)}$	$0.864_{(0.028)}$	$0.729_{(0.039)}$	$0.573_{(0.012)}$	$0.869_{(0.050)}$	$0.868_{(0.061)}$	$0.798_{(0.025)}$	$0.663_{(0.018)}$	0.773
Contextpred[28]	$0.840_{(0.009)}$	$0.877_{(0.026)}$	$0.732_{(0.017)}$	$0.609_{(0.013)}$	$0.882_{(0.065)}$	$0.857_{(0.053)}$	$0.806_{(0.012)}$	$0.714_{(0.018)}$	0.790
Infomax$_{sup}$[28]	$0.839_{(0.037)}$	$0.873_{(0.037)}$	$0.754_{(0.100)}$	$0.622_{(0.008)}$	$0.864_{(0.065)}$	$0.864_{(0.065)}$	$0.826_{(0.016)}$	$0.713_{(0.009)}$	0.794
Edgepred$_{sup}$[28]	$0.847_{(0.024)}$	$0.859_{(0.032)}$	$0.745_{(0.090)}$	$0.620_{(0.007)}$	$0.890_{(0.058)}$	$0.852_{(0.067)}$	$0.829_{(0.021)}$	$0.721_{(0.011)}$	0.795
Masking$_{sup}$[28]	$0.824_{(0.021)}$	$0.859_{(0.020)}$	$0.796_{(0.079)}$	$0.606_{(0.005)}$	$0.888_{(0.041)}$	$0.872_{(0.051)}$	$0.827_{(0.021)}$	$0.715_{(0.007)}$	0.799
Contextpred$_{sup}$[28]	$\underline{0.855}_{(0.023)}$	$0.875_{(0.011)}$	$0.802_{(0.079)}$	$0.620_{(0.009)}$	$0.885_{(0.053)}$	$0.859_{(0.055)}$	$\underline{0.840}_{(0.023)}$	$0.724_{(0.015)}$	0.807
GraphLoG[83]	$0.830_{(0.014)}$	$0.846_{(0.008)}$	$0.667_{(0.021)}$	$0.615_{(0.013)}$	$0.871_{(0.054)}$	$0.850_{(0.080)}$	$0.796_{(0.025)}$	$0.677_{(0.019)}$	0.769
GraphCL[87]	$0.825_{(0.018)}$	$0.887_{(0.019)}$	$0.691_{(0.065)}$	$0.587_{(0.026)}$	$0.875_{(0.048)}$	$0.821_{(0.066)}$	$0.805_{(0.017)}$	$0.696_{(0.023)}$	0.774
JOAO[86]	$0.826_{(0.029)}$	$0.879_{(0.020)}$	$0.741_{(0.047)}$	$\underline{0.640}_{(0.010)}$	$0.861_{(0.066)}$	$0.837_{(0.058)}$	$0.823_{(0.022)}$	$0.711_{(0.014)}$	0.790
GROVER[58]	$0.840_{(0.030)}$	$0.887_{(0.006)}$	$0.874_{(0.048)}$	$0.638_{(0.005)}$	$0.892_{(0.044)}$	$\underline{0.876}_{(0.038)}$	$0.838_{(0.017)}$	$0.696_{(0.014)}$	0.818
3DInfomax[64]	$0.811_{(0.048)}$	$0.877_{(0.014)}$	$\underline{0.887}_{(0.033)}$	$0.585_{(0.017)}$	$0.880_{(0.054)}$	$0.866_{(0.047)}$	$0.805_{(0.032)}$	$0.716_{(0.013)}$	0.804
GraphMVP[42]	$0.818_{(0.012)}$	$0.860_{(0.034)}$	$0.719_{(0.044)}$	$0.584_{(0.026)}$	$0.865_{(0.061)}$	$0.820_{(0.066)}$	$0.799_{(0.018)}$	$0.689_{(0.010)}$	0.769
MolFormer[59]	$0.791_{(0.015)}$	$0.866_{(0.023)}$	$0.810_{(0.059)}$	$0.578_{(0.011)}$	$0.806_{(0.080)}$	$0.651_{(0.063)}$	$0.764_{(0.022)}$	$0.687_{(0.006)}$	0.744
ImageMol[89]	$0.786_{(0.012)}$	$0.881_{(0.034)}$	$0.885_{(0.044)}$	$0.625_{(0.026)}$	$0.839_{(0.061)}$	$0.874_{(0.066)}$	$0.816_{(0.018)}$	$0.710_{(0.010)}$	0.802
GEM[16]	$\mathbf{0.857}_{(0.013)}$	$\underline{0.895}_{(0.020)}$	$0.905_{(0.022)}$	$0.621_{(0.009)}$	$\underline{0.894}_{(0.039)}$	$0.863_{(0.049)}$	$0.832_{(0.016)}$	$\underline{0.733}_{(0.016)}$	$\underline{0.825}$
GraphMAE[27]	$\mathbf{0.857}_{(0.019)}$	$0.878_{(0.032)}$	$0.748_{(0.023)}$	$0.597_{(0.006)}$	$0.881_{(0.062)}$	$0.853_{(0.050)}$	$0.801_{(0.019)}$	$0.691_{(0.020)}$	0.788
MoleBERT[79]	$0.843_{(0.031)}$	$0.851_{(0.022)}$	$0.797_{(0.074)}$	$0.615_{(0.010)}$	$0.887_{(0.046)}$	$0.868_{(0.051)}$	$0.832_{(0.021)}$	$0.720_{(0.009)}$	0.802
KPGT	$\underline{0.855}_{(0.011)}$	$\mathbf{0.908}_{(0.010)}$	$\mathbf{0.946}_{(0.022)}$	$\mathbf{0.649}_{(0.009)}$	$\mathbf{0.905}_{(0.028)}$	$\mathbf{0.889}_{(0.047)}$	$\mathbf{0.848}_{(0.013)}$	$\mathbf{0.746}_{(0.002)}$	**0.843**

Supplementary Table 7: The RMSE performance of different methods on the regression datasets under the transfer learning setting. AVG represents the averaging results over all the datasets. The numbers in brackets are the standard deviations over three independent runs. The best result for each dataset is marked in bold and the second-best result is underlined.

	FreeSolv	ESOL	Lipo	**AVG**
Infomax[72]	$4.119_{(0.974)}$	$1.462_{(0.076)}$	$0.978_{(0.076)}$	2.186
Edgepred[24]	$3.849_{(0.950)}$	$2.272_{(0.213)}$	$1.030_{(0.024)}$	2.384
Masking[28]	$3.646_{(0.947)}$	$2.100_{(0.040)}$	$1.063_{(0.028)}$	2.270
Contextpred[28]	$3.141_{(0.905)}$	$1.349_{(0.069)}$	$0.969_{(0.076)}$	1.820
Infomax_{sup}[28]	$3.017_{(0.964)}$	$1.238_{(0.038)}$	$0.729_{(0.037)}$	1.661
Edgepred_{sup}[28]	$2.889_{(0.877)}$	$1.133_{(0.158)}$	$0.707_{(0.004)}$	1.576
Masking_{sup}[28]	$3.210_{(0.876)}$	$1.387_{(0.007)}$	$0.725_{(0.033)}$	1.774
Contextpred_{sup}[28]	$3.105_{(0.701)}$	$1.477_{(0.038)}$	$0.754_{(0.032)}$	1.779
GraphLoG[83]	$4.174_{(1.077)}$	$2.335_{(0.073)}$	$1.104_{(0.024)}$	2.537
GraphCL[87]	$4.014_{(1.361)}$	$1.835_{(0.111)}$	$0.945_{(0.024)}$	2.264
JOAO[86]	$3.466_{(1.114)}$	$1.771_{(0.053)}$	$0.933_{(0.027)}$	2.056
GROVER[58]	$2.991_{(1.052)}$	$0.928_{(0.027)}$	$0.752_{(0.010)}$	1.557
3DInfomax[64]	$2.919_{(0.243)}$	$1.906_{(0.246)}$	$1.045_{(0.040)}$	1.957
GraphMVP[42]	$2.532_{(0.247)}$	$1.937_{(0.147)}$	$0.990_{(0.024)}$	1.819
MolFormer[59]	$\underline{2.322}_{(0.500)}$	$\underline{0.821}_{(0.056)}$	$0.673_{(0.006)}$	$\underline{1.272}$
ImageMol[89]	$2.634_{(0.720)}$	$1.869_{(0.919)}$	$0.765_{(0.028)}$	1.785
GEM[16]	$2.389_{(0.474)}$	$\mathbf{0.803}_{(0.042)}$	$\underline{0.663}_{(0.006)}$	1.285
GraphMAE[27]	$3.023_{(0.779)}$	$1.378_{(0.028)}$	$0.746_{(0.014)}$	1.716
MoleBERT[79]	$2.801_{(0.602)}$	$1.185_{(0.083)}$	$0.690_{(0.023)}$	1.559
KPGT	$\mathbf{2.121}_{(0.837)}$	$\mathbf{0.803}_{(0.008)}$	$\mathbf{0.600}_{(0.010)}$	**1.175**

Supplementary Table 8: Prediction performance on the datasets from the TDC benchmark of KPGT and the best baseline methods provided by the leaderboards of the TDC benchmark. The numbers in brackets are the standard deviations over five independent runs. The best result for each dataset is marked in bold. Abbreviations: deep learning based method (DL), machine learning based method (ML).

Dataset description				Current best baseline			KPGT	
Group	Dataset	Task	Metric	Method	Type	Score	Score	Rank
Absorption	Caco2	Regression	MAE	BaseBoosting	ML	$0.285_{(0.005)}$	**0.284**$_{(0.009)}$	1st
	HIA	Classification	AUROC	RFStacker	ML	**0.988**$_{(0.002)}$	$0.982_{(0.004)}$	2nd
	Pgp	Classification	AUROC	ZairaChem	ML	$0.935_{(0.006)}$	**0.938**$_{(0.004)}$	1st
	Bioav	Classification	AUROC	SimGCN	DL	$0.748_{(0.033)}$	**0.750**$_{(0.022)}$	1st
	Lipo	Regression	MAE	Chemprop-RDKit	DL	$0.466_{(0.006)}$	**0.446**$_{(0.016)}$	1st
	AqSol	Regression	MAE	Chemprop-RDKit	DL	$0.762_{(0.004)}$	**0.714**$_{(0.011)}$	1st
Distribution	BBB	Classification	AUROC	LRE	DL	**0.962**$_{(0.003)}$	$0.908_{(0.005)}$	6th
	PPBR	Regression	MAE	Chemprop	DL	$7.811_{(0.163)}$	**7.684**$_{(0.250)}$	1st
	VDss	Regression	Spearman's r	Basic ML	ML	$0.627_{(0.010)}$	**0.633**$_{(0.016)}$	1st
Metabolism	CYP2C9$_{inh}$	Classification	AUPRC	ZairaChem	ML	$0.786_{(0.004)}$	**0.797**$_{(0.006)}$	1st
	CYP2D6$_{inh}$	Classification	AUPRC	Chemprop-RDKit	DL	$0.672_{(0.008)}$	**0.724**$_{(0.008)}$	1st
	CYP3A4$_{inh}$	Classification	AUPRC	ZairaChem	ML	$0.875_{(0.002)}$	**0.894**$_{(0.004)}$	1st
	CYP2C9$_{sub}$	Classification	AUPRC	ZairaChem	ML	$0.441_{(0.033)}$	**0.450**$_{(0.044)}$	1st
	CYP2D6$_{sub}$	Classification	AUPRC	Contextpred	DL	$0.736_{(0.024)}$	**0.737**$_{(0.016)}$	1st
	CYP3A4$_{sub}$	Classification	AUPRC	CNN	DL	$0.662_{(0.031)}$	**0.730**$_{(0.023)}$	1st
Excretion	HalfLife	Regression	Spearman's r	Euclia ML model	ML	**0.547**$_{(0.032)}$	$0.531_{(0.030)}$	3rd
	CL-Hepa	Regression	Spearman's r	Basic ML	ML	**0.440**$_{(0.003)}$	$0.424_{(0.019)}$	6th
	CL-Micro	Regression	Spearman's r	RFStacker	ML	$0.625_{(0.002}$	**0.637**$_{(0.010)}$	1st
Toxicity	LD50	Regression	MAE	BaseBoosting	ML	$0.552_{(0.009)}$	**0.545**$_{(0.010)}$	1st
	hERG	Classification	AUROC	SimGCN	DL	**0.874**$_{(0.014)}$	$0.847_{(0.024)}$	3rd
	Ames	Classification	AUROC	ZairaChem	ML	**0.871**$_{(0.002)}$	$0.868_{(0.003)}$	2nd
	DILI	Classification	AUROC	ZairaChem	ML	$0.925_{(0.005)}$	**0.929**$_{(0.013)}$	1st

Supplementary Table 9: Prediction performance on the datasets from the MoleculeACE benchmark of KPGT and the best baseline method provided by the leaderboard of the MoleculeACE benchmark under the MoleculeACE$_{All}$ setting, measured in terms of RMSE. The best result for each dataset is marked in bold. Abbreviations: deep learning based method (DL), machine learning based method (ML).

Dataset	Current best baseline			KPGT	
	Method	Type	Score	Score	Rank
CHEMBL1862_Ki	GROVER	DL	0.668	**0.633**	1st
CHEMBL1871_Ki	SVM$_{ECFP}$	ML	0.668	**0.605**	1st
CHEMBL2034_Ki	GROVER	DL	0.680	**0.679**	1st
CHEMBL204_Ki	SVM$_{ECFP}$	ML	0.705	**0.666**	1st
CHEMBL2047_EC50	GraphMAE	DL	**0.578**	0.588	2nd
CHEMBL214_Ki	GROVER	DL	0.663	**0.652**	1st
CHEMBL2147_Ki	SVM$_{ECFP}$	ML	0.612	**0.587**	1st
CHEMBL218_EC50	RF$_{MACCS}$	ML	0.666	**0.625**	1st
CHEMBL219_Ki	GROVER	DL	0.737	**0.718**	1st
CHEMBL228_Ki	GROVER	DL	0.690	**0.669**	1st
CHEMBL231_Ki	GROVER	DL	0.649	**0.610**	1st
CHEMBL233_Ki	GROVER	DL	0.707	**0.691**	1st
CHEMBL234_Ki	SVM$_{ECFP}$	ML	0.637	**0.606**	1st
CHEMBL235_EC50	RF$_{ECFP}$	ML	0.637	**0.624**	1st
CHEMBL236_Ki	SVM$_{ECFP}$	ML	0.692	**0.655**	1st
CHEMBL237_EC50	SVM$_{ECFP}$	ML	0.760	**0.716**	1st
CHEMBL237_Ki	GROVER	DL	**0.660**	0.678	2nd
CHEMBL238_Ki	GBM$_{ECFP}$	ML	0.611	**0.537**	1st
CHEMBL239_EC50	SVM$_{ECFP}$	ML	0.681	**0.644**	1st
CHEMBL244_Ki	GROVER	DL	0.710	**0.698**	1st
CHEMBL262_Ki	RF$_{ECFP}$	ML	0.703	**0.627**	1st
CHEMBL264_Ki	SVM$_{ECFP}$	ML	0.583	**0.574**	1st
CHEMBL2835_Ki	RF$_{ECFP}$	ML	0.410	**0.373**	1st
CHEMBL287_Ki	GROVER	DL	0.732	**0.706**	1st
CHEMBL2971_Ki	GBM$_{ECFP}$	ML	0.606	**0.571**	1st
CHEMBL3979_EC50	GBM$_{ECFP}$	ML	0.686	**0.669**	1st
CHEMBL4005_Ki	SVM$_{ECFP}$	ML	**0.550**	0.559	2nd
CHEMBL4203_Ki	MoleBERT	DL	**0.820**	0.830	2nd
CHEMBL4616_EC50	SVM$_{ECFP}$	ML	0.589	**0.587**	1st
CHEMBL4792_Ki	SVM$_{ECFP}$	ML	0.675	**0.619**	1st

Supplementary Table 10: Prediction performance on the datasets from the MoleculeACE benchmark of KPGT and the best baseline methods provided by the leaderboards of the MoleculeACE benchmark under the MoleculeACE$_{Cliff}$ setting, measured in terms of RMSE. The best result for each dataset is marked in bold. Abbreviations: deep learning based method (DL), machine learning based method (ML).

Dataset	Current best baseline			KPGT	
	Method	Type	Score	Score	Rank
CHEMBL1862_Ki	SVM$_{ECFP}$	ML	0.634	**0.633**	1st
CHEMBL1871_Ki	GROVER	DL	**0.694**	0.701	2nd
CHEMBL2034_Ki	SVM$_{ECFP}$	ML	0.842	**0.806**	1st
CHEMBL204_Ki	SVM$_{PHYSCHEM}$	ML	0.777	**0.747**	1st
CHEMBL2047_EC50	Edgepred	DL	0.659	**0.654**	1st
CHEMBL214_Ki	GROVER	DL	**0.699**	0.705	2nd
CHEMBL2147_Ki	SVM$_{ECFP}$	ML	0.700	**0.694**	1st
CHEMBL218_EC50	RF$_{MACCS}$	ML	0.733	**0.701**	1st
CHEMBL219_Ki	GROVER	DL	0.749	**0.727**	1st
CHEMBL228_Ki	GBM$_{ECFP}$	ML	**0.755**	0.759	2nd
CHEMBL231_Ki	GraphLoG	DL	**0.768**	0.774	3rd
CHEMBL233_Ki	GROVER	DL	0.751	**0.731**	1st
CHEMBL234_Ki	SVM$_{ECFP}$	ML	0.683	**0.646**	1st
CHEMBL235_EC50	RF$_{ECFP}$	ML	0.761	**0.759**	1st
CHEMBL236_Ki	SVM$_{ECFP}$	ML	0.836	**0.793**	1st
CHEMBL237_EC50	SVM$_{MACCS}$	ML	0.842	**0.785**	1st
CHEMBL237_Ki	Masking	DL	0.707	**0.706**	1st
CHEMBL238_Ki	GBM$_{ECFP}$	ML	0.696	**0.657**	1st
CHEMBL239_EC50	SVM$_{ECFP}$	ML	**0.731**	0.746	2nd
CHEMBL244_Ki	GROVER	DL	0.829	**0.820**	1st
CHEMBL262_Ki	SVM$_{ECFP}$	ML	0.655	**0.601**	1st
CHEMBL264_Ki	SVM$_{ECFP}$	ML	0.668	**0.642**	1st
CHEMBL2835_Ki	GEM	DL	0.714	**0.666**	1st
CHEMBL287_Ki	GraphMAE	DL	0.746	**0.729**	1st
CHEMBL2971_Ki	GBM$_{ECFP}$	ML	**0.739**	0.792	4th
CHEMBL3979_EC50	SVM$_{ECFP}$	ML	0.727	**0.723**	1st
CHEMBL4005_Ki	SVM$_{ECFP}$	ML	**0.647**	0.673	2nd
CHEMBL4203_Ki	EdgePred$_{Sup}$	DL	**0.915**	1.114	14th
CHEMBL4616_EC50	SVM$_{ECFP}$	ML	0.618	**0.583**	1st
CHEMBL4792_Ki	Contextpred	DL	0.734	**0.709**	1st

Supplementary Table 11: Top-20 predictions of KPGT to be potential inhibitors against HPK1 with known evidence found from previous studies in the literature.

ID	Drugbank ID	Name	Prediction (pIC50)	Evidence
1	DB01268	Sunitinib	8.324	The Ki of Sunitinib against HPK1 was measured to be about 16 nM by competition binding assays[12]
2	DB12141	Gilteritinib	7.659	Not found
3	DB12500	Fedratinib	7.644	The Ki of Fedratinib against HPK1 was measured to be about 9 nM by homogeneous time-resolved fluorescence (HTRF) assay[7]
4	DB09063	Ceritinib	7.41	The Ki of Ceritinib against HPK1 was measured to be about $2e^{-5}$ uM by LanthaScreen assay[6]
5	DB12010	Fostamatinib	7.313	The Kd of Fostamatinib against HPK1 was measured to be about 72 nM by binding constant for the HPK1 kinase domain[36]
6	DB09073	Palbociclib	7.244	Not found
7	DB11828	Neratinib	7.207	The Kd of Neratinib against HPK1 was measured to be about 16 nM by binding constant for the HPK1 kinase domain[12]
8	DB12001	Abemaciclib	7.199	The Ki of Abemaciclib against HPK1 was measured to be about 0.03963 uM by LanthaScreen time resolved fluorescence resonance energy transfer (TR-FRET)[66]
9	DB11963	Dacomitinib	7.168	The Ki of Dacomitinib against HPK1 was measured to be about $4.76e^{-4}$ uM by LanthaScreen assay[6]
10	DB11652	Tucatinib	7.147	The Ki of Tucatinib against HPK1 was measured to be about 0.0347 uM by TR-FRET assay[66]
11	DB14840	Ripretinib	7.096	Not found
12	DB15442	Trilaciclib	7.071	Not found
13	DB12332	Rucaparib	7.067	Not found
14	DB05294	Vandetanib	7.056	The Kd of Neratinib against HPK1 was measured to be about 5500 nM by binding constant for the HPK1 kinase domain[12]
15	DB15685	Selpercatinib	7.050	Not found
16	DB08916	Afatinib	7.031	The IC50 of Afatinib against HPK1 was measured to be about 6.3 nM by TR-FRET assay[33]
17	DB09079	Nintedanib	7.013	The IC50 of Nintedanib against HPK1 was measured to be about 45 nM by TR-FRET assay[33]
18	DB08881	Vemurafenib	7.007	The Ki of Vemurafenib against HPK1 was measured to be about $1.13e^{-4}$ uM by LanthaScreen assay[6]
19	DB09335	Alatrofloxacin	6.994	Not found
20	DB15456	Vericiguat	6.990	Not found

Supplementary Table 12: Top-20 predictions of KPGT to be potential inhibitors against FGFR1 kinase with known evidence found from previous studies in the literature.

ID	Drugbank ID	Name	Prediction (pIC50)	Evidence
1	DB12147	Erdafitinib	8.964	Erdafitinib inhibited FGFR1 in TR-FRET assay with IC50 value of 1.2 nmol/L[53]
2	DB08901	Ponatinib	8.009	Ponatinib inhibited FGFR1 in ELISA assay with IC50 value of 0.7 nmol/L[19]
3	DB08881	Vemurafenib	7.978	Vemurafenib inhibited FGFR1 in kinase inhibition assay with inhibition rate of 24%[90]
4	DB08875	Cabozantinib	7.640	Cabozantinib inhibited FGFR1 with IC50 value of 11.3 nM[41]
5	DB11886	Infigratinib	7.559	Infigratinib binds to FGFR1 with IC50 value of 1.1 nM[34]
6	DB09079	Nintedanib	7.371	Ninetedanib inhibited FGFR1 in ELISA assay with value of 0.06 uM[76]
7	DB12267	Brigatinib	7.046	Not found
8	DB15102	Pemigatinib	7.044	Pemigatinib inhibited FGFR1 in FRET assay with IC50 value of 0.4 nM[78]
9	DB06589	Pazopanib	6.938	Pazopanib inhibited FGFR1 with IC50 value of 140 nM[25]
10	DB09078	Lenvatinib	6.934	Lenvatinib inhibited FGFR1 in tyrosine kinase assay with IC50 value of 46 nM[48]
11	DB14840	Ripretinib	6.757	Not found
12	DB11986	Entrectinib	6.754	Entretinib inhibited FGFR1 with IC50 value of 1 uM[49]
13	DB11718	Encorafenib	6.689	Not found
14	DB12010	Fostamatinib	6.687	Fostamatinib can act as inhibitor of FGFR1[57]
15	DB08865	Crizotinib	6.652	Crizotinib inhibited FGFR1 in ELISA with IC50 value of 1000 nM[43]
16	DB11979	Elagolix	6.526	Not found
17	DB06626	Axitinib	6.516	Axitinib inhibited FGFR1 with Ki value of 56 nM[35]
18	DB11817	Baricitinib	6.514	Not found
19	DB13874	Enasidenib	6.478	Not found
20	DB08877	Ruxolitinib	6.472	Not found

Supplementary Table 13: Ablation studies of KPGT. Several modified frameworks based on KPGT with specific restrictions are introduced, including KPGT-Pretrain (without pre-training), KPGT-KN (without the knowledge nodes), KPGT-PE (without the path encoding module), KPGT-DE (without the distance encoding module), KPGT-LG (replacing molecular line graph with the molecular graph used in Graphormer), KPGT-LiGhT+Graphormer (replacing the backbone model LiGhT with Graphormer), and KPGT-LiGhT+GIN (replacing the backbone model LiGhT with GIN). The average results on the classification and regression datasets from the first benchmarking test in the Results section were reported, measured in terms of AUROC and RMSE, respectively. The numbers in brackets are the standard deviations across three independent runs. The best results are marked in bold.

Method	Classification dataset **AVG** (AUROC)	Regression dataset **AVG** (RMSE)
KPGT-LiGhT+GIN	$0.817_{(0.009)}$	$1.229_{(0.257)}$
KPGT-LiGhT+Graphormer	$0.822_{(0.007)}$	$1.260_{(0.081)}$
KPGT-LG	$0.824_{(0.006)}$	$1.250_{(0.303)}$
KPGT-PE	$0.822_{(0.007)}$	$1.281_{(0.254)}$
KPGT-DE	$0.823_{(0.012)}$	$1.306_{(0.144)}$
KPGT-KN	$0.819_{(0.008)}$	$1.287_{(0.304)}$
KPGT-Pretrain	$0.792_{(0.001)}$	$1.469_{(0.302)}$
KPGT	**0.829**$_{(0.011)}$	**1.162**$_{(0.197)}$

Supplementary Table 14: The 200 molecular descriptors employed in KPGT.

BalabanJ	BertzCT	Chi0	Chi0n	Chi0v
Chi1	Chi1n	Chi1v	Chi2n	Chi2v
Chi3n	Chi3v	Chi4n	Chi4v	EState_VSA1
EState_VSA10	EState_VSA11	EState_VSA2	EState_VSA3	EState_VSA4
EState_VSA5	EState_VSA6	EState_VSA7	EState_VSA8	EState_VSA9
ExactMolWt	FpDensityMorgan1	FpDensityMorgan2	FpDensityMorgan3	FractionCSP3
HallKierAlpha	HeavyAtomCount	HeavyAtomMolWt	Ipc	Kappa1
Kappa2	Kappa3	LabuteASA	MaxAbsEStateIndex	MaxAbsPartialCharge
MaxEStateIndex	MaxPartialCharge	MinAbsEStateIndex	MinAbsPartialCharge	MinEStateIndex
MinPartialCharge	MolLogP	MolMR	MolWt	NHOHCount
NOCount	NumAliphaticCarbocycles	NumAliphaticHeterocycles	NumAliphaticRings	NumAromaticCarbocycles
NumAromaticHeterocycles	NumAromaticRings	NumHAcceptors	NumHDonors	NumHeteroatoms
NumRadicalElectrons	NumRotatableBonds	NumSaturatedCarbocycles	NumSaturatedHeterocycles	NumSaturatedRings
NumValenceElectrons	PEOE_VSA1	PEOE_VSA10	PEOE_VSA11	PEOE_VSA12
PEOE_VSA13	PEOE_VSA14	PEOE_VSA2	PEOE_VSA3	PEOE_VSA4
PEOE_VSA5	PEOE_VSA6	PEOE_VSA7	PEOE_VSA8	PEOE_VSA9
RingCount	SMR_VSA1	SMR_VSA10	SMR_VSA2	SMR_VSA3
SMR_VSA4	SMR_VSA5	SMR_VSA6	SMR_VSA7	SMR_VSA8
SMR_VSA9	SlogP_VSA1	SlogP_VSA10	SlogP_VSA11	SlogP_VSA12
SlogP_VSA2	SlogP_VSA3	SlogP_VSA4	SlogP_VSA5	SlogP_VSA6
SlogP_VSA7	SlogP_VSA8	SlogP_VSA9	TPSA	VSA_EState1
VSA_EState10	VSA_EState2	VSA_EState3	VSA_EState4	VSA_EState5
VSA_EState6	VSA_EState7	VSA_EState8	VSA_EState9	fr_Al_COO
fr_Al_OH	fr_Al_OH_noTert	fr_ArN	fr_Ar_COO	fr_Ar_N
fr_Ar_NH	fr_Ar_OH	fr_COO	fr_COO2	fr_C_O
fr_C_O_noCOO	fr_C_S	fr_HOCCN	fr_Imine	fr_NH0
fr_NH1	fr_NH2	fr_N_O	fr_Ndealkylation1	fr_Ndealkylation2
fr_Nhpyrrole	fr_SH	fr_aldehyde	fr_alkyl_carbamate	fr_alkyl_halide
fr_allylic_oxid	fr_amide	fr_amidine	fr_aniline	fr_aryl_methyl
fr_azide	fr_azo	fr_barbitur	fr_benzene	fr_benzodiazepine
fr_bicyclic	fr_diazo	fr_dihydropyridine	fr_epoxide	fr_ester
fr_ether	fr_furan	fr_guanido	fr_halogen	fr_hdrzine
fr_hdrzone	fr_imidazole	fr_imide	fr_isocyan	fr_isothiocyan
fr_ketone	fr_ketone_Topliss	fr_lactam	fr_lactone	fr_methoxy
fr_morpholine	fr_nitrile	fr_nitro	fr_nitro_arom	fr_nitro_arom_nonortho
fr_nitroso	fr_oxazole	fr_oxime	fr_para_hydroxylation	fr_phenol
fr_phenol_noOrthoHbond	fr_phos_acid	fr_phos_ester	fr_piperdine	fr_piperzine
fr_priamide	fr_prisulfonamd	fr_pyridine	fr_quatN	fr_sulfide
fr_sulfonamd	fr_sulfone	fr_term_acetylene	fr_tetrazole	fr_thiazole
fr_thiocyan	fr_thiophene	fr_unbrch_alkane	fr_urea	qed

Supplementary References

1. Tox21 challenge (2017), `http://tripod.nih.gov/tox21/challenge/`
2. Adasme, M.F., Linnemann, K.L., Bolz, S.N., Kaiser, F., Salentin, S., Haupt, V.J., Schroeder, M.: Plip 2021: Expanding the scope of the protein–ligand interaction profiler to dna and rna. Nucleic acids research **49**(W1), W530–W534 (2021)
3. Boral, N., Ghosh, P., Goswami, A., Bhattacharyya, M.: Accountable prediction of drug admet properties with molecular descriptors. bioRxiv pp. 2022–06 (2022)
4. Breiman, L.: Bagging predictors. Machine learning **24**, 123–140 (1996)
5. Broccatelli, F., Carosati, E., Neri, A., Frosini, M., Goracci, L., Oprea, T.I., Cruciani, G.: A novel approach for predicting p-glycoprotein (abcb1) inhibition using molecular interaction fields. Journal of medicinal chemistry **54**(6), 1740–1751 (2011)
6. Bryan Chan, B.D., et al.: Naphthyridines as inhibitors of hpk1. U.S. Patent WO2018183956A1 (2018)
7. Bryan Chan, J.D., et al.: Isoquinolines as inhibitors of hpk1. U.S. Patent 2018183964A1 (2018)
8. Burchett, P.: Colorrefinement + weighted ensemble lgbm. https://github.com/parkerburchett/TDC-DeepLearning/blob/main/modeling/CYP%20Weighted%20Model.ipynb (2021)
9. Carbon-Mangels, M., Hutter, M.C.: Selecting relevant descriptors for classification by bayesian estimates: a comparison with decision trees and support vector machines approaches for disparate data sets. Molecular informatics **30**(10), 885–895 (2011)
10. Chithrananda, S., Grand, G., Ramsundar, B.: Chemberta: Large-scale self-supervised pretraining for molecular property prediction. arXiv preprint arXiv:2010.09885 (2020)
11. Cristianini, N., Shawe-Taylor, J., et al.: An introduction to support vector machines and other kernel-based learning methods. Cambridge university press (2000)
12. Davis, M.I., Hunt, J.P., Herrgard, S., Ciceri, P., Wodicka, L.M., Pallares, G., Hocker, M., Treiber, D.K., Zarrinkar, P.P.: Comprehensive analysis of kinase inhibitor selectivity. Nature biotechnology **29**(11), 1046–1051 (2011)
13. Delaney, J.S.: Esol: estimating aqueous solubility directly from molecular structure. Journal of chemical information and computer sciences **44**(3), 1000–1005 (2004)
14. Di, L., Keefer, C., Scott, D.O., Strelevitz, T.J., Chang, G., Bi, Y.A., Lai, Y., Duckworth, J., Fenner, K., Troutman, M.D., et al.: Mechanistic insights from comparing intrinsic clearance values between human liver microsomes and hepatocytes to guide drug design. European journal of medicinal chemistry **57**, 441–448 (2012)
15. Durant, J.L., Leland, B.A., Henry, D.R., Nourse, J.G.: Reoptimization of mdl keys for use in drug discovery. Journal of chemical information and computer sciences **42**(6), 1273–1280 (2002)
16. Fang, X., Liu, L., Lei, J., He, D., Zhang, S., Zhou, J., Wang, F., Wu, H., Wang, H.: Geometry-enhanced molecular representation learning for property prediction. Nature Machine Intelligence **4**(2), 127–134 (2022)
17. Fix, E., Hodges, J.L.: Discriminatory analysis: nonparametric discrimination: consistency properties. Nearest Neighbor (NN) Norms: NN Pattern Classification Techniques pp. 32–39 (1991)
18. Friedman, J.H.: Greedy function approximation: a gradient boosting machine. Annals of statistics pp. 1189–1232 (2001)
19. Gao, Y., Zhang, P., Cui, A., Ye, D.Y., Xiang, M., Chu, Y.: Discovery and anti-inflammatory evaluation of benzothiazepinones (btzs) as novel non-atp competitive inhibitors of glycogen synthase kinase-3β (gsk-3β). Bioorganic & Medicinal Chemistry **26**(20), 5479–5493 (2018)
20. Gaulton, A., Bellis, L.J., Bento, A.P., Chambers, J., Davies, M., Hersey, A., Light, Y., McGlinchey, S., Michalovich, D., Al-Lazikani, B., et al.: Chembl: a large-scale bioactivity database for drug discovery. Nucleic acids research **40**(D1), D1100–D1107 (2012)
21. Gaulton, A., et al.: The chembl database in 2017. Nucleic acids research **45**(D1), D945–D954 (2017)
22. Gayvert, K.M., Madhukar, N.S., Elemento, O.: A data-driven approach to predicting successes and failures of clinical trials. Cell chemical biology **23**(10), 1294–1301 (2016)
23. Gilmer, J., Schoenholz, S.S., Riley, P.F., Vinyals, O., Dahl, G.E.: Neural message passing for quantum chemistry. In: International conference on machine learning. pp. 1263–1272. PMLR (2017)
24. Hamilton, W.L., Ying, Z., Leskovec, J.: Inductive representation learning on large graphs. In: NeurIPS 2017. pp. 1024–1034 (2017)
25. Harris, P.A., Boloor, A., Cheung, M., Kumar, R., Crosby, R.M., Davis-Ward, R.G., Epperly, A.H., Hinkle, K.W., Hunter III, R.N., Johnson, J.H., et al.: Discovery of 5-[[4-[(2, 3-dimethyl-2 h-indazol-6-yl) methylamino]-2-pyrimidinyl] amino]-2-methyl-benzenesulfonamide (pazopanib), a novel and potent vascular endothelial growth factor receptor inhibitor. Journal of medicinal chemistry **51**(15), 4632–4640 (2008)
26. Hou, T., Wang, J., Zhang, W., Xu, X.: Adme evaluation in drug discovery. 7. prediction of oral absorption by correlation and classification. Journal of chemical information and modeling **47**(1), 208–218 (2007)
27. Hou, Z., Liu, X., Cen, Y., Dong, Y., Yang, H., Wang, C., Tang, J.: Graphmae: Self-supervised masked graph autoencoders. In: Proceedings of the 28th ACM SIGKDD Conference on Knowledge Discovery and Data Mining. pp. 594–604 (2022)

28. Hu, W., Liu, B., Gomes, J., Zitnik, M., Liang, P., Pande, V.S., Leskovec, J.: Strategies for pre-training graph neural networks. In: ICLR 2020 (2020)
29. Huang, D., Chowdhuri, S.R., Li, A., Li, A., Agrawal, A., Gano, K., Zhu, A.: A unified system for molecular property predictions: Oloren chemengine and its applications (2022)
30. Huang, K., Fu, T., Gao, W., Zhao, Y., Roohani, Y., Leskovec, J., Coley, C.W., Xiao, C., Sun, J., Zitnik, M.: Therapeutics data commons: Machine learning datasets and tasks for drug discovery and development. Proceedings of Neural Information Processing Systems, NeurIPS Datasets and Benchmarks (2021)
31. Huang, K., Fu, T., Gao, W., Zhao, Y., Roohani, Y., Leskovec, J., Coley, C.W., Xiao, C., Sun, J., Zitnik, M.: Artificial intelligence foundation for therapeutic science. Nature Chemical Biology (2022)
32. Huang, K., Fu, T., Glass, L.M., Zitnik, M., Xiao, C., Sun, J.: Deeppurpose: a deep learning library for drug–target interaction prediction. Bioinformatics **36**(22-23), 5545–5547 (2020)
33. Jing Li, Z.W., XU, S.: Pyrrolo [2, 3-b] pyridines or pyrrolo [2, 3-b] pyrazines as hpk1 inhibitor and the use thereof. U.S. Patent WO2019238067A1 (2019)
34. Kang, C.: Infigratinib: first approval. Drugs **81**(11), 1355–1360 (2021)
35. Kania, R.S.B., et al.: Indazole compounds and pharmaceutical compositions for inhibiting protein kinases, and methods for their use. U.S. Patent US20040220248A1 (2004)
36. Karaman, M.W., Herrgard, S., Treiber, D.K., Gallant, P., Atteridge, C.E., Campbell, B.T., Chan, K.W., Ciceri, P., Davis, M.I., Edeen, P.T., et al.: A quantitative analysis of kinase inhibitor selectivity. Nature biotechnology **26**(1), 127–132 (2008)
37. Kimber, T.B., Gagnebin, M., Volkamer, A.: Maxsmi: maximizing molecular property prediction performance with confidence estimation using smiles augmentation and deep learning. Artificial Intelligence in the Life Sciences **1**, 100014 (2021)
38. Kipf, T.N., Welling, M.: Semi-supervised classification with graph convolutional networks. arXiv preprint arXiv:1609.02907 (2016)
39. Kuhn, M., Letunic, I., Jensen, L.J., Bork, P.: The sider database of drugs and side effects. Nucleic acids research **44**(D1), D1075–D1079 (2016)
40. Lee, W.H., Millman, S., Desai, N., Srivatsa, M., Liu, C.: Neuralfp: out-of-distribution detection using fingerprints of neural networks. In: 2020 25th International Conference on Pattern Recognition (ICPR). pp. 9561–9568. IEEE (2021)
41. Li, J., An, B., Song, X., Zhang, Q., Chen, C., Wei, S., Fan, R., Li, X., Zou, Y.: Design, synthesis and biological evaluation of novel 2, 4-diaryl pyrimidine derivatives as selective egfrl858r/t790m inhibitors. European Journal of Medicinal Chemistry **212**, 113019 (2021)
42. Liu, S., Wang, H., Liu, W., Lasenby, J., Guo, H., Tang, J.: Pre-training molecular graph representation with 3d geometry. arXiv preprint arXiv:2110.07728 (2021)
43. Liu, Y., Jin, S., Peng, X., Lu, D., Zeng, L., Sun, Y., Ai, J., Geng, M., Hu, Y.: Pyridazinone derivatives displaying highly potent and selective inhibitory activities against c-met tyrosine kinase. European Journal of Medicinal Chemistry **108**, 322–333 (2016)
44. Lombardo, F., Jing, Y.: In silico prediction of volume of distribution in humans. extensive data set and the exploration of linear and nonlinear methods coupled with molecular interaction fields descriptors. Journal of Chemical Information and Modeling **56**(10), 2042–2052 (2016)
45. Ma, C.Y., Yang, S.Y., Zhang, H., Xiang, M.L., Huang, Q., Wei, Y.Q.: Prediction models of human plasma protein binding rate and oral bioavailability derived by using ga–cg–svm method. Journal of pharmaceutical and biomedical analysis **47**(4-5), 677–682 (2008)
46. Malchow, S., Korepanova, A., Panchal, S.C., McClure, R.A., Longenecker, K.L., Qiu, W., Zhao, H., Cheng, M., Guo, J., Klinge, K.L., et al.: The hpk1 inhibitor a-745 verifies the potential of modulating t cell kinase signaling for immunotherapy. ACS Chemical Biology **17**(3), 556–566 (2022)
47. Martins, I.F., Teixeira, A.L., Pinheiro, L., Falcao, A.O.: A bayesian approach to in silico blood-brain barrier penetration modeling. Journal of chemical information and modeling **52**(6), 1686–1697 (2012)
48. Matsui, J., Yamamoto, Y., Funahashi, Y., Tsuruoka, A., Watanabe, T., Wakabayashi, T., Uenaka, T., Asada, M.: E7080, a novel inhibitor that targets multiple kinases, has potent antitumor activities against stem cell factor producing human small cell lung cancer h146, based on angiogenesis inhibition. International journal of cancer **122**(3), 664–671 (2008)
49. Menichincheri, M., Ardini, E., Magnaghi, P., Avanzi, N., Banfi, P., Bossi, R., Buffa, L., Canevari, G., Ceriani, L., Colombo, M., et al.: Discovery of entrectinib: a new 3-aminoindazole as a potent anaplastic lymphoma kinase (alk), c-ros oncogene 1 kinase (ros1), and pan-tropomyosin receptor kinases (pan-trks) inhibitor. Journal of medicinal chemistry **59**(7), 3392–3408 (2016)
50. Mobley, D.L., Guthrie, J.P.: Freesolv: a database of experimental and calculated hydration free energies, with input files. Journal of computer-aided molecular design **28**(7), 711–720 (2014)
51. Moret, M., Grisoni, F., Katzberger, P., Schneider, G.: Perplexity-based molecule ranking and bias estimation of chemical language models. Journal of chemical information and modeling **62**(5), 1199–1206 (2022)

52. Obach, R.S., Lombardo, F., Waters, N.J.: Trend analysis of a database of intravenous pharmacokinetic parameters in humans for 670 drug compounds. Drug Metabolism and Disposition **36**(7), 1385–1405 (2008)
53. Perera, T.P., Jovcheva, E., Mevellec, L., Vialard, J., De Lange, D., Verhulst, T., Paulussen, C., Van De Ven, K., King, P., Freyne, E., et al.: Discovery and pharmacological characterization of jnj-42756493 (erdafitinib), a functionally selective small-molecule fgfr family inhibitorpreclinical characterization of erdafitinib. Molecular cancer therapeutics **16**(6), 1010–1020 (2017)
54. Podlewska, S., Kafel, R.: Metstabon—online platform for metabolic stability predictions. International journal of molecular sciences **19**(4), 1040 (2018)
55. Richard, A.M., et al.: Toxcast chemical landscape: paving the road to 21st century toxicology. Chemical research in toxicology **29**(8), 1225–1251 (2016)
56. Rogers, D., Hahn, M.: Extended-connectivity fingerprints. Journal of chemical information and modeling **50**(5), 742–754 (2010)
57. Rolf, M.G., Curwen, J.O., Veldman-Jones, M., Eberlein, C., Wang, J., Harmer, A., Hellawell, C.J., Braddock, M.: In vitro pharmacological profiling of r406 identifies molecular targets underlying the clinical effects of fostamatinib. Pharmacology research & perspectives **3**(5), e00175 (2015)
58. Rong, Y., Bian, Y., Xu, T., Xie, W., Wei, Y., Huang, W., Huang, J.: Self-supervised graph transformer on large-scale molecular data. In: NeurIPS 2020 (2020)
59. Ross, J., Belgodere, B., Chenthamarakshan, V., Padhi, I., Mroueh, Y., Das, P.: Large-scale chemical language representations capture molecular structure and properties. Nature Machine Intelligence **4**(12), 1256–1264 (2022). https://doi.org/10.1038/s42256-022-00580-7
60. Scarlat, A.: Maccs keys + automl. https://github.com/scarlat1/AcuteToxicityLD50 (2021)
61. Schmidhuber, J., Hochreiter, S., et al.: Long short-term memory. Neural Comput **9**(8), 1735–1780 (1997)
62. Shen, W.X., Zeng, X., Zhu, F., Wang, Y.L., Qin, C., Tan, Y., Jiang, Y.Y., Chen, Y.Z.: Out-of-the-box deep learning prediction of pharmaceutical properties by broadly learned knowledge-based molecular representations. Nature Machine Intelligence **3**(4), 334–343 (2021)
63. Sorkun, M.C., Khetan, A., Er, S.: Aqsoldb, a curated reference set of aqueous solubility and 2d descriptors for a diverse set of compounds. Scientific data **6**(1), 1–8 (2019)
64. Stärk, H., Beaini, D., Corso, G., Tossou, P., Dallago, C., Günnemann, S., Liò, P.: 3d infomax improves gnns for molecular property prediction. arXiv preprint arXiv:2110.04126 (2021)
65. Subramanian, G., Ramsundar, B., Pande, V., Denny, R.A.: Computational modeling of β-secretase 1 (bace-1) inhibitors using ligand based approaches. Journal of chemical information and modeling **56**(10), 1936–1949 (2016)
66. Sushant Malhotra, C.S., et al.: Spirocyclic 2,3-dihydro-7-azaindole compounds and uses thereof. U.S. Patent WO2020061377A1 (2020)
67. van Tilborg, D., Alenicheva, A., Grisoni, F.: Exposing the limitations of molecular machine learning with activity cliffs. Journal of Chemical Information and Modeling **62**(23), 5938–5951 (2022). https://doi.org/10.1021/acs.jcim.2c01073, `https://doi.org/10.1021/acs.jcim.2c01073`, pMID: 36456532
68. Todeschini, R., Gramatica, P.: 3d qsar in drug design. ed. H. Kubinyi, G. Folkers, and YC Martin," Kluwer/ESCOM **2** (1998)
69. Turon, G., Hlozek, J., Woodland, J., Chibale, K., Duran-Frigola, M.: First fully-automated ai/ml virtual screening cascade implemented at a drug discovery centre in africa. bioRxiv pp. 2022–12 (2022)
70. Veith, H., Southall, N., Huang, R., James, T., Fayne, D., Artemenko, N., Shen, M., Inglese, J., Austin, C.P., Lloyd, D.G., et al.: Comprehensive characterization of cytochrome p450 isozyme selectivity across chemical libraries. Nature biotechnology **27**(11), 1050–1055 (2009)
71. Velickovic, P., Cucurull, G., Casanova, A., Romero, A., Lio, P., Bengio, Y., et al.: Graph attention networks. stat **1050**(20), 10–48550 (2017)
72. Velickovic, P., Fedus, W., Hamilton, W.L., Liò, P., Bengio, Y., Hjelm, R.D.: Deep graph infomax. In: ICLR 2019. OpenReview.net (2019)
73. Walters, W.P., Murcko, M.A.: Prediction of 'drug-likeness'. Advanced drug delivery reviews **54**(3), 255–271 (2002)
74. Wang, N.N., Dong, J., Deng, Y.H., Zhu, M.F., Wen, M., Yao, Z.J., Lu, A.P., Wang, J.B., Cao, D.S.: Adme properties evaluation in drug discovery: prediction of caco-2 cell permeability using a combination of nsga-ii and boosting. Journal of chemical information and modeling **56**(4), 763–773 (2016)
75. Wang, S., Sun, H., Liu, H., Li, D., Li, Y., Hou, T.: Admet evaluation in drug discovery. 16. predicting herg blockers by combining multiple pharmacophores and machine learning approaches. Molecular pharmaceutics **13**(8), 2855–2866 (2016)
76. Wang, X., Chen, Z., Tong, L., Tan, S., Zhou, W., Peng, T., Han, K., Ding, J., Xie, H., Xu, Y.: Naphthalimides exhibit in vitro antiproliferative and antiangiogenic activities by inhibiting both topoisomerase ii (topo ii) and receptor tyrosine kinases (rtks). European Journal of Medicinal Chemistry **65**, 477–486 (2013)
77. Wenlock, M., Tomkinson, N.: Experimental in vitro dmpk and physicochemical data on a set of publicly disclosed compounds (2015)

78. Wu, L., Zhang, C., He, C., Qian, D., Lu, L., Sun, Y., Xu, M., Zhuo, J., Liu, P.C., Klabe, R., et al.: Discovery of pemigatinib: a potent and selective fibroblast growth factor receptor (fgfr) inhibitor. Journal of Medicinal Chemistry **64**(15), 10666–10679 (2021)
79. Xia, J., Zhao, C., Hu, B., Gao, Z., Tan, C., Liu, Y., Li, S., Li, S.Z.: Mole-bert: Rethinking pre-training graph neural networks for molecules. In: The Eleventh International Conference on Learning Representations (2022)
80. Xiong, Z., et al.: Pushing the boundaries of molecular representation for drug discovery with the graph attention mechanism. Journal of medicinal chemistry **63**(16), 8749–8760 (2019)
81. Xu, C., Cheng, F., Chen, L., Du, Z., Li, W., Liu, G., Lee, P.W., Tang, Y.: In silico prediction of chemical ames mutagenicity. Journal of chemical information and modeling **52**(11), 2840–2847 (2012)
82. Xu, K., Hu, W., Leskovec, J., Jegelka, S.: How powerful are graph neural networks? In: ICLR 2019 (2019)
83. Xu, M., Wang, H., Ni, B., Guo, H., Tang, J.: Self-supervised graph-level representation learning with local and global structure. In: ICML 2021. vol. 139, pp. 11548–11558 (2021)
84. Xu, Y., Dai, Z., Chen, F., Gao, S., Pei, J., Lai, L.: Deep learning for drug-induced liver injury. Journal of chemical information and modeling **55**(10), 2085–2093 (2015)
85. Yang, K., et al.: Analyzing learned molecular representations for property prediction. Journal of chemical information and modeling **59**(8), 3370–3388 (2019)
86. You, Y., Chen, T., Shen, Y., Wang, Z.: Graph contrastive learning automated. In: ICML 2021. vol. 139, pp. 12121–12132 (2021)
87. You, Y., Chen, T., Sui, Y., Chen, T., Wang, Z., Shen, Y.: Graph contrastive learning with augmentations. In: NeurIPS 2020 (2020)
88. Yuan, H., Yu, H., Wang, J., Li, K., Ji, S.: On explainability of graph neural networks via subgraph explorations. In: International conference on machine learning. pp. 12241–12252. PMLR (2021)
89. Zeng, X., Xiang, H., Yu, L., Wang, J., Li, K., Nussinov, R., Cheng, F.: Accurate prediction of molecular properties and drug targets using a self-supervised image representation learning framework. Nature Machine Intelligence **4**(11), 1004–1016 (2022)
90. Zhang, Z.X., Jin, W.J., Yang, S., Ji, C.L.: Braf kinase inhibitor exerts anti-tumor activity against breast cancer cells via inhibition of fgfr2. American journal of cancer research **6**(5), 1040 (2016)
91. Zhu, H., Martin, T.M., Ye, L., Sedykh, A., Young, D.M., Tropsha, A.: Quantitative structure- activity relationship modeling of rat acute toxicity by oral exposure. Chemical research in toxicology **22**(12), 1913–1921 (2009)
