# Supplementary material for: A knowledge-guided pre-training framework for improving molecular representation learning
Source: Nat Commun. 2023 Nov 21;14:7568. doi: 10.1038/s41467-023-43214-1 (PMC10663446; doi:10.1038/s41467-023-43214-1)
Supplement: Supplementary file 3 — Reporting Summary [file 41467_2023_43214_MOESM3_ESM.pdf]

nature portfolio

Corresponding author(s): Dan Zhao, Jianyang Zeng

Last updated by author(s): Oct 11, 2023

Reporting Summary

Nature Portfolio wishes to improve the reproducibility of the work that we publish. This form provides structure for consistency and transparency in reporting. For further information on Nature Portfolio policies, see our Editorial Policies and the Editorial Policy Checklist.

Statistics

For all statistical analyses, confirm that the following items are present in the figure legend, table legend, main text, or Methods section.

n/a	Confirmed	
☐	☒	The exact sample size (n) for each experimental group/condition, given as a discrete number and unit of measurement
☐	☒	A statement on whether measurements were taken from distinct samples or whether the same sample was measured repeatedly
☒	☐	The statistical test(s) used AND whether they are one- or two-sided<br>*Only common tests should be described solely by name; describe more complex techniques in the Methods section.*
☒	☐	A description of all covariates tested
☒	☐	A description of any assumptions or corrections, such as tests of normality and adjustment for multiple comparisons
☒	☐	A full description of the statistical parameters including central tendency (e.g. means) or other basic estimates (e.g. regression coefficient) AND variation (e.g. standard deviation) or associated estimates of uncertainty (e.g. confidence intervals)
☒	☐	For null hypothesis testing, the test statistic (e.g. F, t, r) with confidence intervals, effect sizes, degrees of freedom and P value noted<br>*Give P values as exact values whenever suitable.*
☒	☐	For Bayesian analysis, information on the choice of priors and Markov chain Monte Carlo settings
☒	☐	For hierarchical and complex designs, identification of the appropriate level for tests and full reporting of outcomes
☐	☒	Estimates of effect sizes (e.g. Cohen's d, Pearson's r), indicating how they were calculated

Our web collection on statistics for biologists contains articles on many of the points above.

Software and code

Policy information about availability of computer code

Data collection	python (3.7.13), torch (1.10.0), dgl (0.7.2), rdkit (2021.09.2), numpy(1.21.5), pandas (1.3.5)
Data analysis	The model was implemented using python (3.7.13) with torch (1.10.0) and dgl (0.7.2). The docking results were visualized using Pymol (2.5.0). All docking experiments were conducted using AutoDock Vina (1.2.3). The Protein-Ligand Interaction Profiler web tool cby visiting the following link: https://plip-tool.biotec.tu-dresden.de/plip-web/plip/index. The source codes of KPGT are available on Github repository at https://github.com/lihan97/KPGT.

For manuscripts utilizing custom algorithms or software that are central to the research but not yet described in published literature, software must be made available to editors and reviewers. We strongly encourage code deposition in a community repository (e.g. GitHub). See the Nature Portfolio guidelines for submitting code & software for further information.

Data

Policy information about availability of data

All manuscripts must include a data availability statement. This statement should provide the following information, where applicable:

- Accession codes, unique identifiers, or web links for publicly available datasets
- A description of any restrictions on data availability
- For clinical datasets or third party data, please ensure that the statement adheres to our policy

The processed ChEMBL29 dataset and the datasets from the first benchmarking tests in the Results section can be accessed via Figshare (DOI: 10.6084/m9.figshare.19914811). The datasets from the TDC benchmark platform are available at https://tdcommons.ai/benchmark/overview/. The datasets from the

MoleculeACE benchmark are available at https://github.com/molML/MoleculeACE. The HPK1 and FGFR1 datasets can be accessed via Figshare (DOI: 10.6084/m9.figshare.24290899). The FDA dataset is available at https://go.drugbank.com/releases/5-1-10/downloads/approved-structure-links. The reference protein-ligand complex structures for HPK1 and FGFR1 used in this study are available in the Protein Data Bank (PDB) under accession codes 7SIU and 5A4C, respectively. Preliminary results from this study have been reported in a conference proceedings version. Source data are provided with this paper.

Research involving human participants, their data, or biological material

Policy information about studies with human participants or human data. See also policy information about sex, gender (identity/presentation), and sexual orientation and race, ethnicity and racism.

Reporting on sex and gender	N/A
Reporting on race, ethnicity, or other socially relevant groupings	N/A
Population characteristics	N/A
Recruitment	N/A
Ethics oversight	N/A

Note that full information on the approval of the study protocol must also be provided in the manuscript.

Field-specific reporting

Please select the one below that is the best fit for your research. If you are not sure, read the appropriate sections before making your selection.

☒ Life sciences ☐ Behavioural & social sciences ☐ Ecological, evolutionary & environmental sciences

For a reference copy of the document with all sections, see nature.com/documents/nr-reporting-summary-flat.pdf

Life sciences study design

All studies must disclose on these points even when the disclosure is negative.

Sample size	No sample size calculation was performed. The datasets employed in the benchmarking tests within the Results section were derived from previous literature and have been extensively utilized for evaluating models in molecular property prediction. Consequently, we are confident in the adequacy of the sample sizes for these datasets. Moreover, the consistent performane across different datasets also demonstrates the sample sizes are sufficent.
Data exclusions	For the HPK1 and FGFR1 datasets, We excluded the molecules that overlapped with the pre-training data employed by KPGT in the prediction tests.
Replication	We repeat the same tests with different random seeds to verify the reproducibility of the experimental findings. The detailed can be found in the section titled "Statistics & Reproducibility".
Randomization	We used different random seeds for dataset splitting and parameter initialization in independent runs.
Blinding	The investigators were blinded to group allocation.

Reporting for specific materials, systems and methods

We require information from authors about some types of materials, experimental systems and methods used in many studies. Here, indicate whether each material, system or method listed is relevant to your study. If you are not sure if a list item applies to your research, read the appropriate section before selecting a response.

Materials & experimental systems

n/a	Involved in the study	
☒	☐	Antibodies
☒	☐	Eukaryotic cell lines
☒	☐	Palaeontology and archaeology
☒	☐	Animals and other organisms
☒	☐	Clinical data
☒	☐	Dual use research of concern
☒	☐	Plants

Methods

n/a	Involved in the study	
☒	☐	ChIP-seq
☒	☐	Flow cytometry
☒	☐	MRI-based neuroimaging
